# Transcriptomic resources for prairie grass (*Bromus catharticus*): expressed transcripts, tissue-specific genes, and identification and validation of EST-SSR markers

**DOI:** 10.1186/s12870-021-03037-y

**Published:** 2021-06-07

**Authors:** Ming Sun, Zhixiao Dong, Jian Yang, Wendan Wu, Chenglin Zhang, Jianbo Zhang, Junming Zhao, Yi Xiong, Shangang Jia, Xiao Ma

**Affiliations:** 1 Department of Grassland Science, College of Animal Science and Technology, Sichuan Agricultural University, Chengdu, 611130 Sichuan China; 2 College of Grassland Science and Technology, China Agricultural University, Beijing, 100193 China; 3 Sichuan Academy of Grassland Science, Chengdu, 611731 Sichuan China; 4 Key Laboratory of Pratacultural Science, Beijing Municipality, China Agricultural University, Beijing, 100193 China

**Keywords:** *Bromus catharticus*, Transcriptome sequencing, Differentially expressed genes, Marker development, Diversity analysis

## Abstract

**Background:**

Prairie grass (*Bromus catharticus*) is a typical cool-season forage crop with high biomass production and fast growth rate during winter and spring. However, its genetic research and breeding has remained stagnant due to limited available genomic resources. The aim of this study was to generate large-scale genomic data using high-throughput transcriptome sequencing, and perform a preliminary validation of EST-SSR markers of *B. catharticus*.

**Results:**

Eleven tissue samples including seeds, leaves, and stems were collected from a new high-yield strain of prairie grass BCS1103. A total of 257,773 unigenes were obtained, of which 193,082 (74.90%) were annotated. Comparison analysis between tissues identified 1803, 3030, and 1570 genes specifically and highly expressed in seed, leaf, and stem, respectively. A total of 37,288 EST-SSRs were identified from unigene sequences, and more than 80,000 primer pairs were designed. We synthesized 420 primer pairs and selected 52 ones with high polymorphisms to estimate genetic diversity and population structure in 24 *B. catharticus* accessions worldwide. Despite low diversity indicated by an average genetic distance of 0.364, the accessions from South America and Asia and wild accessions showed higher genetic diversity. Moreover, South American accessions showed a pure ancestry, while Asian accessions demonstrated mixed internal relationships, which indicated a different probability of gene flow. Phylogenetic analysis clustered the studied accessions into four clades, being consistent with phenotypic clustering results. Finally, Mantel analysis suggested the total phenotypic variation was mostly contributed by genetic component. Stem diameter, plant height, leaf width, and biomass yield were significantly correlated with genetic data (*r* > 0.6, *P* < 0.001), and might be used in the future selection and breeding.

**Conclusion:**

A genomic resource was generated that could benefit genetic and taxonomic studies, as well as molecular breeding for *B. catharticus* and its relatives in the future.

**Supplementary Information:**

The online version contains supplementary material available at 10.1186/s12870-021-03037-y.

## Background

*Bromus* L. is a genus distributed widely in the temperate world, which contains approximately 150 C3 grass species, many of which are not taxonomically identified [1, 2]. *Bromus catharticus* is one of the major agricultural grass species, which belongs to the *Ceratochloa* section (hexaploid, 2n = 6x = 42), and has become naturalized in many areas, with facultative cleistogamous reproductive behavior and annual or short-lived perennial growth forms [1]. Due to high biomass yield, fast growth rate during winter and spring, strong adaptability, and the ability to remain green after seed maturation, *B. catharticus* has become more and more popular as a cool-season forage grass in the mountainous and hilly areas of Southwest China [3, 4]. However, as cultivated varieties of *B. catharticus* were mostly developed through hybrid selection and domestication from wild germplasms, its genomic resources are considerably limited, resulting in slow progress on molecular breeding and taxonomy research.

Next generation sequencing technologies provide cutting-edge approaches for high-throughput sequence generation, allowing rapid and comprehensive analyses of genomes and transcripts. Extensive transcriptomic studies have already been applied in various plant biological contexts. Where for instance, comparative transcriptome analysis on different tissues and stages was used to reveal the regulatory modules of tissues development [5–7], or the metabolism of specific biochemical components [8–10], and tissue-specific expression profiling has proved effective in uncovering biological pathways and regulatory networks. Despite general applications of genomic approaches, to date, these have not yet been applied to research on *B. catharticus*. Therefore, it is still necessary to provide an original reference transcriptome profile for *B. catharticus* and its relatives.

Transcriptome sequencing has also been used to identify molecular markers, especially simple sequence repeat (SSR) markers, in many forage grass species, such as *Medicago sativa* [11], *Elymus sibiricus* [12, 13], and *Lolium multiflorum* [14] due to its cost-efficiency. Molecular markers have a great potential in the modern plant breeding process, and have been widely applied to determine genetic diversity, genotype identification, and in marker-assisted selection (MAS) [13, 15]. However, the lack of marker information for molecular phylogeny and genetic structure limited brome (*Bromus* L.) species collection, conservation and utilization, and there are as of yet few available EST sequences of *Bromus* L. in the GenBank database (https://www.ncbi.nlm.nih.gov/). Previously, amplified fragment length polymorphisms (AFLPs) were estimated in 32 South American and one North American *Bromus* genus accessions [2]. RAPD and AFLP analyses on *B. catharticus* showed a narrow genetic basis of varieties from France and Singapore, which provided a reference for the use of molecular marker techniques to select parent genotypes to broaden the genetic basis of these varieties [16]. SSRs and AFLPs were used to compare the correlation between molecular markers and significant genetic variation in *Bromus tectorum*, and SSRs were suggested to be good surrogates for phenotypic traits in population genetic studies of strongly inbred species [17]. Due to a lack of effective molecular markers from genetic data of its own species or genus, SSRs and EST-SSRs (expressed sequence tag-SSRs) from wheat (*Triticum aestivum*) were employed on *Bromus inermis* to evaluate marker transferability, and showed advantages such as co-dominance, stability, and high reproducibility [18]. EST-SSRs have been confirmed to have excellent transferability between different species of the same genus, and even between different genera. Furthermore, transcriptome sequencing (RNA-seq) is useful in the identification and development of a great number of SSR markers, and is faster and more cost effective compared to the traditional SSR development processes [13].

The present study reports a comprehensive transcriptome sequencing of a *B. catharticus* genotype. The major objectives were (a) to generate ESTs from various tissues at different developmental stages via transcriptomic sequencing of *B. catharticus*, and to annotate a *de novo* transcriptome assembly; (b) to compare transcript expression between different tissues and identify tissue-specific genes; (c) to carry out large-scale *in silico* identification of SSRs from transcriptomic data; and (d) to perform genetic diversity and population structure analyses on a set of germplasm accessions combined with phenotypic data. A genomic resource was generated that showed preliminary potential for application in molecular-assisted selection, which may provide a basis for further investigation of the genetics and taxonomy of *Bromus* and its relatives.

## Results

### Illumina sequencing, *de novo* assembly, and functional annotation

**Fig. 1:**
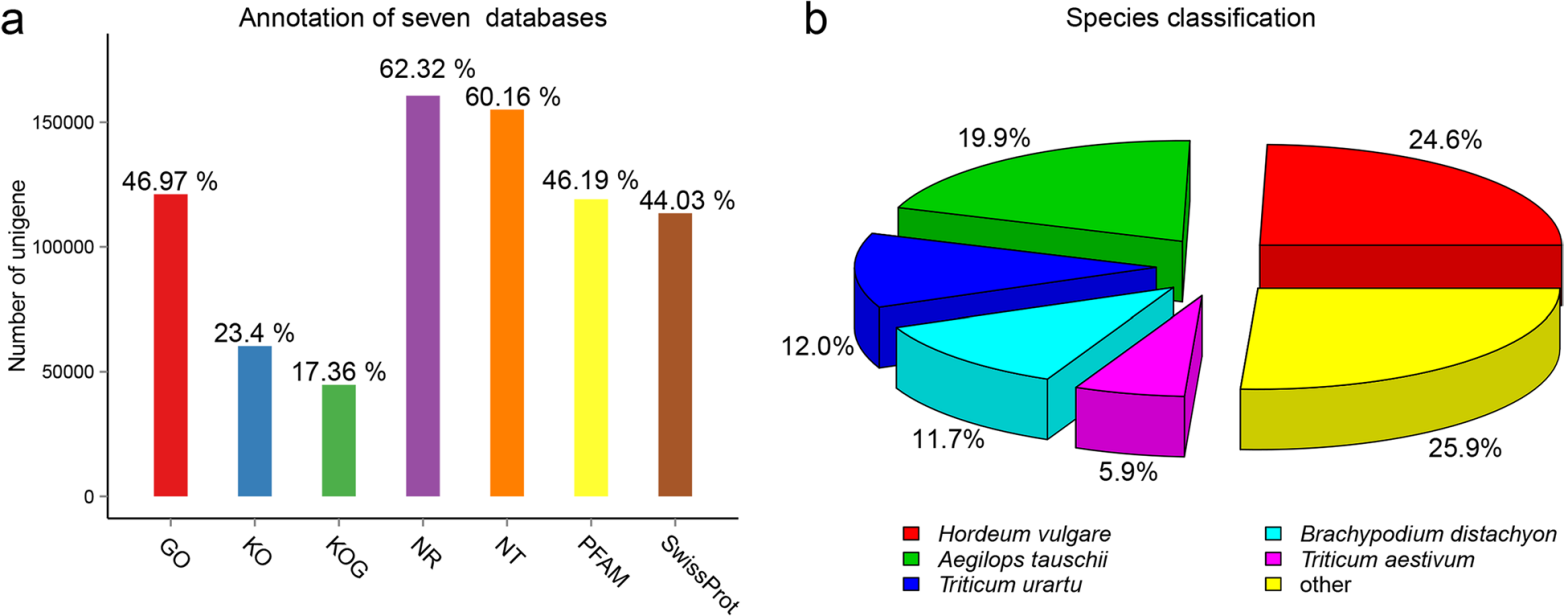
Unigene annotation. **a** Unigenes were annotated in seven databases: Nr, Nt, PFAM, Swiss-Prot, KO, KOG, and GO. **b** Species assignment of all unigenes based on Nr database annotation

Illumina sequencing of eleven libraries for seed, leaf, and stem tissues generated an average of 48,945,309 raw reads and 46,614,613 clean reads, and high-quality clean reads were used for subsequent analyses (Table 1). Transcripts amounting to 450,361 were assembled with an N50 length of 1346 bp and an N90 length of 281 bp, ranging from 201 to14,257 bp with a mean of 766 bp. The transcripts were then clustered into a total of 257,773 unigenes with 1129 bp, 1629 bp, and 531 bp for mean length, N50, and N90, respectively (Table 2 & Fig. S1). The different expression patterns and normal distributions of the density distributions of FPKM in the eleven samples are shown in Fig. S2. A total of 193,082 (74.90%) of the unigenes were annotated against public protein databases, with 160,662 (62.32%), 155,087 (60.16%), 119,080 (46.19%), 113,501 (44.03%), 60,321 (23.4%), 44,775 (17.36%), and 121,098 (46.97%) unigenes having at least one hit from the NCBI Nr, Nt, PFAM, Swiss-Prot, KEGG, KOG, and GO databases, respectively (Fig. 1a). Unigenes amounting to 25,739 (9.98%) were annotated in all seven databases, and 64,691 unigenes (25.1%) did not have any matches, and are potentially novel genes that arose during the evolution of the *B. catharticus* genome. Based on Nr database annotation, compared to more than 700 species, unigenes of *B. catharticus* were shown with the top match of *Hordeum vulgare* (24.6%), followed by *Aegilops tauschii* (19.9%), *Triticum urartu* (12.0%), *Brachypodium distachyon* (11.7%), and *Triticum aestivum* (5.9%; Fig. 1b). GO enrichment analysis of unigenes showed that cellular processes, metabolic processes, and single-organism processes were highly enriched, especially “binding” (66,849) and “catalytic activity” (53,923) in the molecular function category, and “cell” (38,314) and “cell part” (38,293) among cellular components (Fig. S3a). Among the five categories of KEGG pathways, metabolism was the largest, with 11 subgroups and 28,653 genes, including the most significant subgroup of transcription with 7,828 genes (Fig. S3b).

**Table 1:** Summary of transcriptome sequencing data for *B. catharticus*

Sample IDs	Raw reads	Clean reads	Clean bases (G)	Q20 (%)	GC content (%)
S1	49,551,462	47,034,074	7.06	96.27	57.56
S2	44,389,500	41,857,858	6.28	95.96	55.53
S3	47,129,920	44,433,520	6.67	96.23	56.39
F1	50,042,628	47,488,618	7.12	96.30	53.44
F2	56,344,998	53,995,100	8.10	96.19	54.39
L1	41,792,200	39,406,238	5.91	96.26	56.06
L2	46,567,082	44,247,074	6.64	96.31	55.51
L3	48,262,052	46,279,372	6.94	96.42	55.47
T1	46,451,132	44,600,276	6.69	96.17	55.56
T2	53,009,106	50,781,390	7.62	96.14	56.32
T3	54,858,318	52,637,220	7.90	96.29	55.14
Mean	48,945,309	46,614,613	6.99	96.23	55.58

**Table 2:** Statistics of transcripts and unigenes obtained via Trinity assembly

	Min length	Mean length	Median length	Max length	N50	N90	Total nucleotides
Transcript (bp)	201	766	402	14,257	1,346	281	344,828,318
Unigene (bp)	201	1,129	808	14,257	1,629	531	290,927,105

### Analysis of DEGs in tissues of *B. catharticus*

**Fig. 2:**
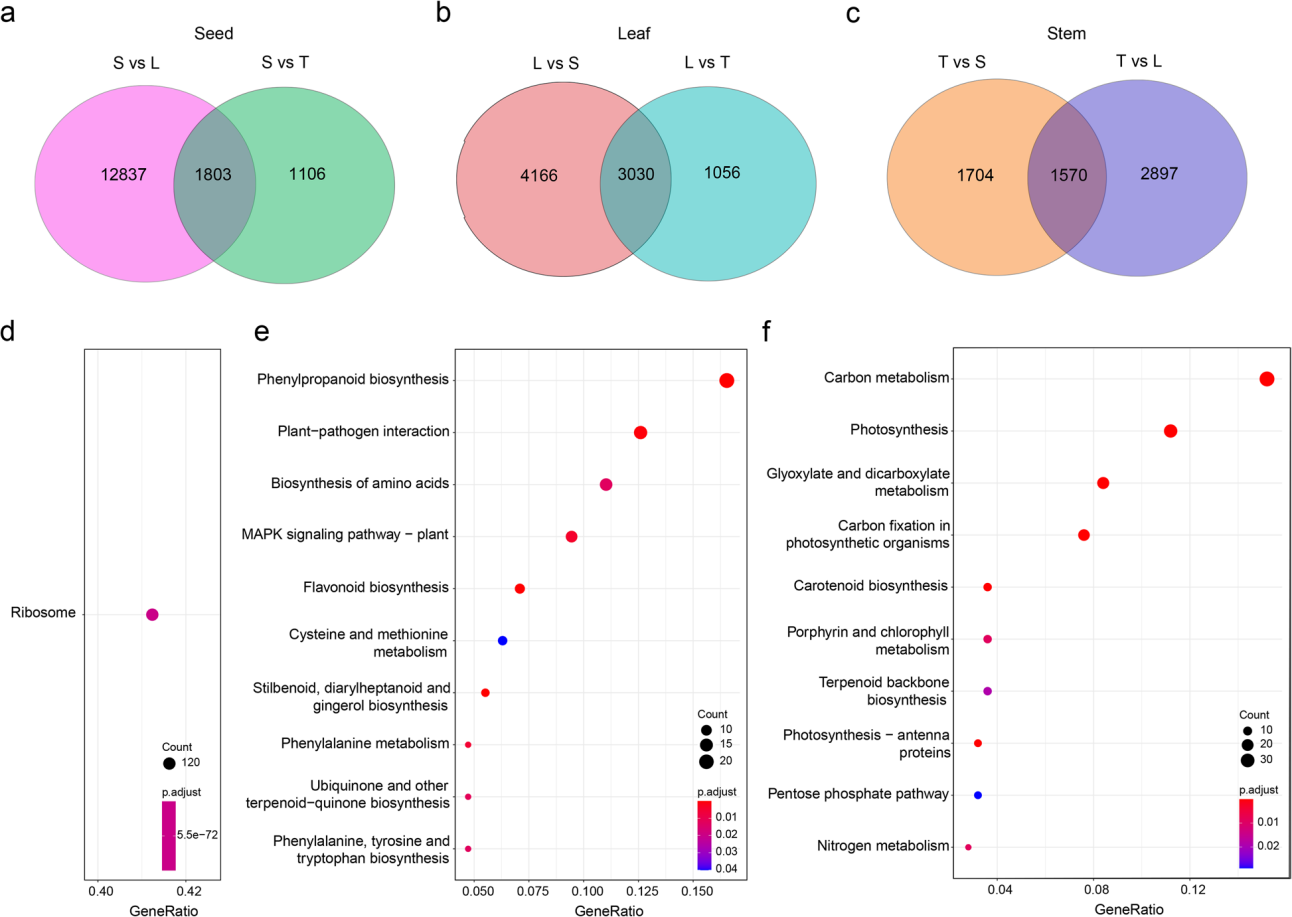
Tissue-specific genes and their KEGG annotation in seeds (termed S, including S1, S2, F1 and F2), leaf (L, including L1, L2, and L3) and stem (T, including T1, T2, and T3). Venn diagrams showed the number of genes specifically expressed in seeds (**a**), leaves (**b**) and stems (**c**). KEGG pathway enrichment diagrams of seed- (**d**), leaf- (**e**) and stem- (**f**) specific genes are shown

**Fig. 3:**
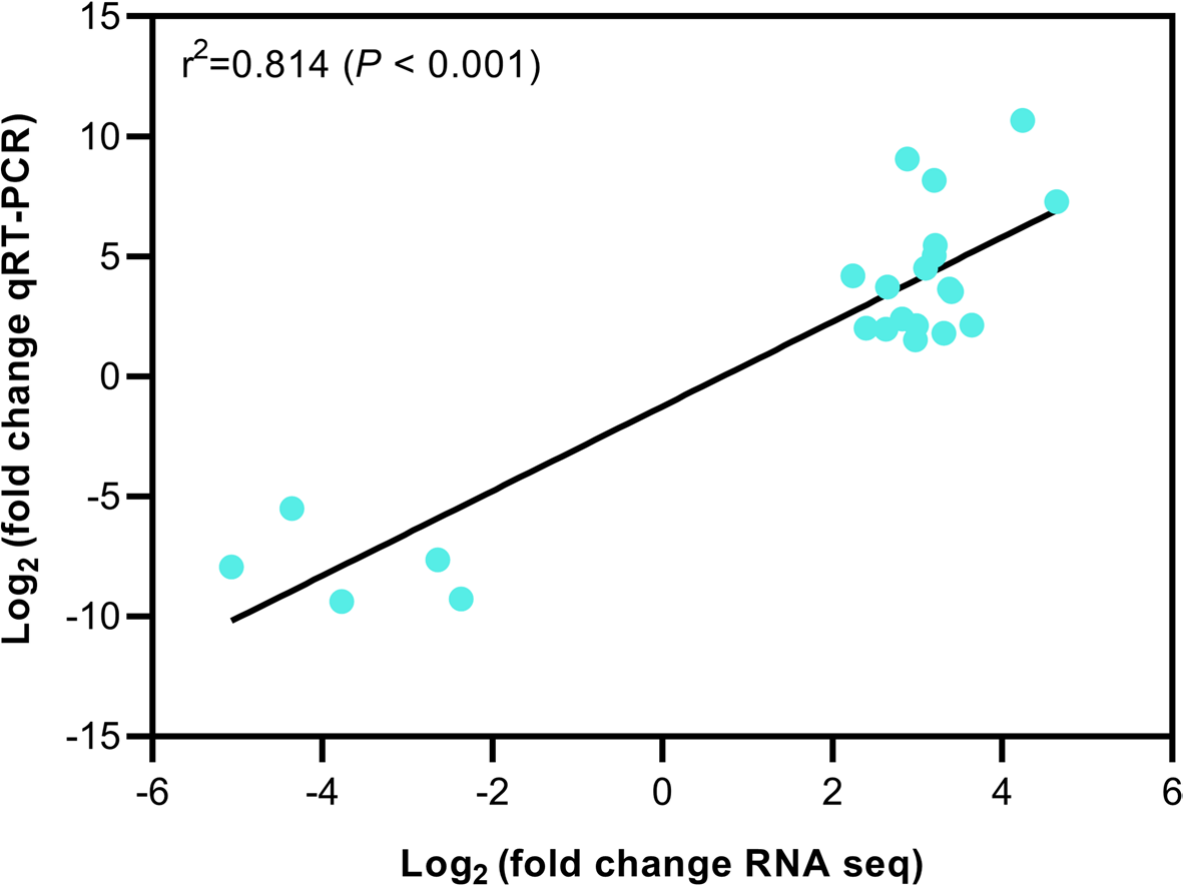
Verification of tissue-specific genes obtained from transcriptome sequencing by qRT-PCR

In order to identify tissue-specific genes, we placed 10 transcriptomic samples into three groups, i.e., group S for seed with S1, S2, F1, and F2, group L for leaf with L1, L2, and L3, and group T for stem with T1, T2 and T3. We conducted a comparative analysis among S, L, and T for DEGs (*P* < 0.05), and determined tissue-specific highly expressed genes in one tissue, but not in the other tissues. We plotted Venn diagrams for the upregulated DEGs in the three pair-wise comparisons (i.e., S vs L, S vs T, and L vs T). A total of 1803 genes were found to be specifically and highly expressed in developing and germinating seeds (overlapping DEGs in “S vs L” and “S vs T,” Fig. 2a), but not in the other two tissues of leaf and stem. And 3,030 and 1,570 genes were specifically and highly expressed in leaves (overlapping DEGs in “L vs S” and “L vs T,” Fig. 2b) and stems (overlapping DEGs in “T vs S” and “T vs L,” Fig. 2c), respectively. We found genes specifically and highly expressed in developing and germinating seeds were significantly enriched in the KEGG pathway of “ribosome” (Fig. 2d). Leaf-specific highly expressed genes were significantly enriched in genes involved in “carbon metabolism”, “photosynthesis”, “glyoxylate and dicarboxylate metabolism”, and “carbon fixation in photosynthetic organisms” (Fig. 2e). Stem-specific highly expressed genes were significantly enriched in genes involved in “phenylpropanoid biosynthesis”, “plant-pathogen interaction”, “amino acid biosynthesis”, and “MAPK signaling pathway” (Fig. 2f). To validate DEGs obtained from RNA-seq, we performed qRT-PCR analysis for 11 selected genes in three tissue samples (F2, L2 and T2), and the result was in good agreement with RNA-seq results (*r*^2^ = 0.814, *P* < 0.001, Fig. 3).

### Identification of EST-SSR markers and genetic diversity

**Fig. 4:**
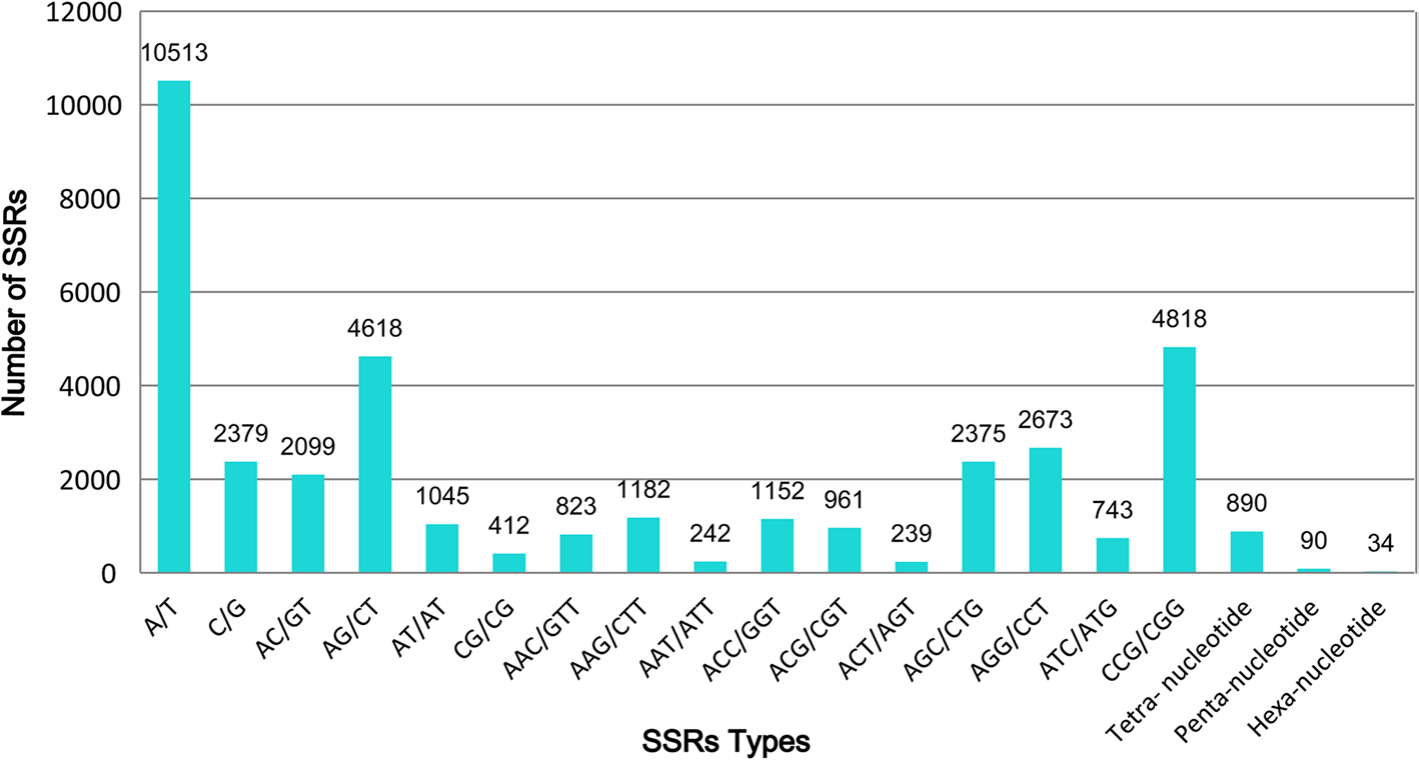
Distribution of different types of EST-SSRs

A total of 37,288 SSRs were identified in 32,370 (14.47%) unigenes, in which more than one SSR was present in 4,238 transcripts. Tri-nucleotide SSR repeats were the most abundant (15,208), followed by mono-nucleotides (12,892) and di-nucleotides (8,174). A or T repeats (10,513) were four or five times more common than C or G repeats (2,379) among mono-nucleotide repeats. CCG/CGG (4,818), AGG/CCT (2,673) and AGC/CTG (2,375) comprised 64.87% of all the tri-nucleotide repeat motifs among SSRs (Fig. 4, Table S1). AAAG/CTTT, ATCCG/ATCGG, and ACCTCC/AGGTGG repeats dominated the tetra-, penta-, and hexa-nucleotide repeats, respectively, although they were rare. Of SSRs, 27,540 were successfully used to design > 80,000 primers; a total of 420 primer pairs were randomly selected to determine polymorphisms and performance, and finally 350 primer pairs were successfully used to amplify DNA fragments across 24 *B. catharticus* accessions. To further validate the sequences of the SSR loci, ten PCR products from nine accessions for two SSR markers were sequenced. In all of the cases, the sequenced alleles from the selected accessions were homologous to the original locus from which the primers were designed, which effectively guarantees acceptable downstream analysis (Fig. S4).

**Fig. 5:**
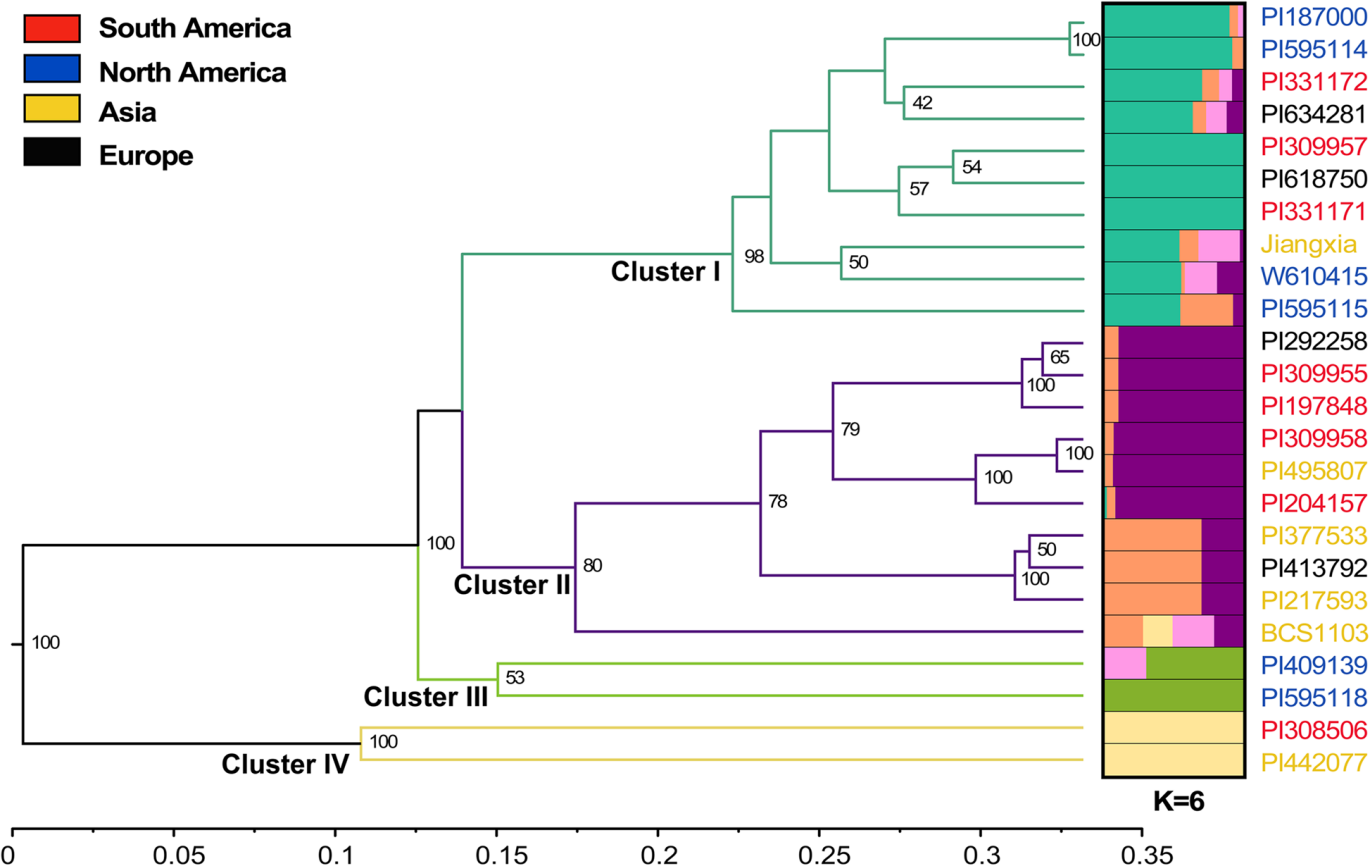
Population structure of 24 *B. cartharticus* accessions based on UPGMA and Bayesian clustering (STRUCTURE, K = 6). All accessions were grouped into four clusters. The accession codes with red, blue, yellow, and black indicated that these accessions were collected from South America, North America, Asia, and Europe, respectively. All accessions colored based on the sample origins were divided into four UPGMA clusters and five STRUCTURE subgroups (represented by five colors: blue-green, purple, orange, green, and light yellow). The numbers beside each branch represent bootstrap values, and only the numbers higher than 40% are presented

We further selected 52 effective primer pairs that were designed based on the di-, tri-, and tetra-nucleotide SSRs with 5–10 repeats (Table S2). The mean PP, PIC, Rp, and MI values were 91.60%, 0.254, 1.14, and 0.67, respectively. Among the three repeat types, tetra-nucleotide SSRs showed relatively higher PIC (0.290), Rp (1.29), and MI (0.73) values, indicating a high discriminatory power. Primers from five-repeat SSRs showed higher PIC values (0.285), while seven-repeat SSRs showed higher Rp (1.29) and MI (0.86) values (Table 3). A total of 152 bands amplified by 52 selected primer pairs were used to estimate Nei’s genetic distance of the 24 accessions. The genetic distance values ranged from 0.008 to 0.790, with an average distance of 0.364. The maximum value (0.790) was observed between accession PI442077 and PI309955, and the minimum coefficient value (0.008) was observed between PI187000 and PI595114. To study the genetic relationship and population structure of *B. catharticus*, UPGMA clustering analysis and STRUCTURE analysis were performed (Fig. 5). Based on maximum likelihood and delta K values (K = 6), the accessions in this study were divided into five STRUCTURE subgroups (represented by five colors: blue-green, purple, orange, green, and light yellow). The result of four UPGMA clusters (Cluster I ~ IV) was consistent with STRUCTURE analysis. Cluster II contained two STRUCTURE subgroups (purple and orange). Both Clusters I and II consisted of 10 accessions, and only two accessions were found in Clusters III and IV, respectively. In addition, 14 accessions (> 50%) had high membership coefficients (Q-value > 80%), including 7, 2, 2, and 3 accessions from South America, Asia, Europe, and North America, respectively (Fig. 5). Genetic diversity in the subpopulation mostly matched the geographical locations. The genetic diversity index (He) among the four continents varied from 0.173 (Europe) to 0.238 (Asia), with an average of 0.209 (Table 4). Additionally, accessions collected from the wild had relatively high genetic diversity (0.259), followed by cultivar materials (0.251), and then materials of uncertain breeding status (0.179).

**Table 3:** Marker parameters calculated for each SSR type based on 52 primer pairs

SSR type		NP	ANF	APF	PP (%)	PIC	Rp	MI
Repeat types	Di-	5	3.20	3.00	95.00%	0.153	0.58	0.45
	Tri-	18	3.22	2.89	89.35%	0.225	1.06	0.64
	Tetra-	29	3.17	2.90	92.41%	0.290	1.29	0.73
Number of repeats	10	1	5.00	5.00	100.00%	0.168	1.00	0.84
	9	1	3.00	3.00	100.00%	0.192	0.67	0.58
	8	2	3.00	2.50	87.50%	0.126	0.46	0.27
	7	6	3.33	3.33	100.00%	0.238	1.29	0.86
	6	15	3.27	2.87	87.22%	0.232	1.06	0.62
	5	27	3.07	2.78	91.85%	0.285	1.23	0.69
Total		52	3.19	2.90	91.60%	0.254	1.14	0.67

*NP* Number of primer pairs, *ANF* Average number of fragments, *APF* Average number of polymorphic fragments, *PP* Percent of polymorphic fragments, *PIC* Polymorphic information content, *Rp* Resolving power, *MI* Marker index

**Table 4:** Genetic diversity estimates for groups based on geographical distribution and breeding status

	Pop	N	Na	Ne	I	He
Geographical distribution	South America	8	1.461	1.398	0.354	0.234
	Asia	6	1.447	1.414	0.358	0.238
	Europe	4	1.086	1.297	0.256	0.173
	North America	6	1.263	1.323	0.285	0.189
	Mean	6	1.314	1.358	0.313	0.209
Breeding status	Cultivar	6	1.520	1.430	0.378	0.251
	Uncertain material	8	1.125	1.308	0.266	0.179
	Wild material	10	1.743	1.435	0.397	0.259
	Mean	8	1.463	1.391	0.347	0.230

*N* Number of accessions, *Na* Number of different alleles, *Ne* Number of effective alleles, *I* Shannon' information index, *He* Expected heterozygosity

### Phenotypic variation and Mantel analysis

**Fig. 6:**
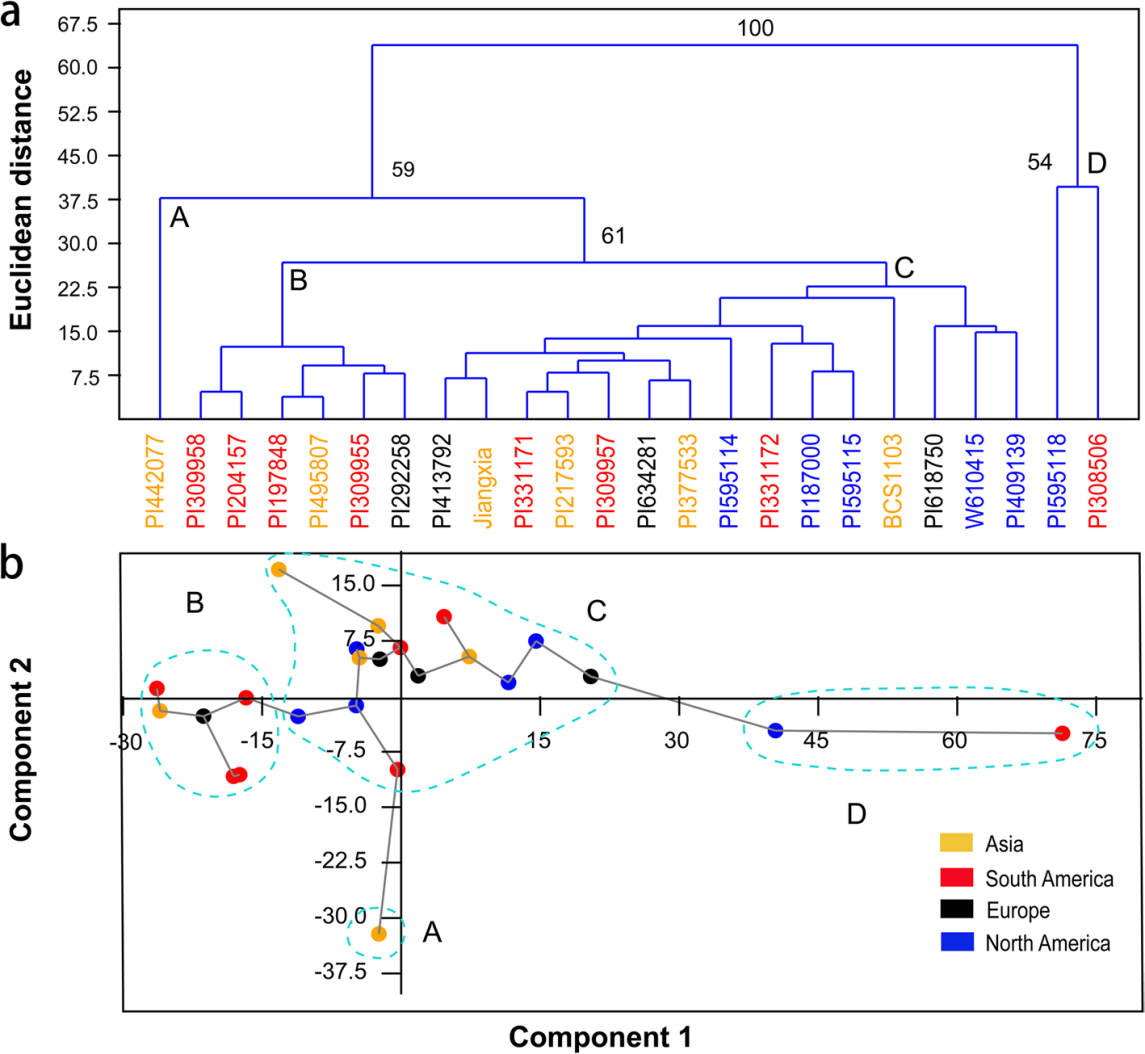
UPGMA cluster (**a**) and PCA (**b**) of 24 *B. catharticus* accessions based on 11 phenotypic traits. The accession codes and dots with red, blue, yellow, and black indicate these accessions were collected from South America, North America, Asia, and Europe, respectively. The letters of A, B, C, and D indicate Group A, Group B, Group C, and Group D, respectively

The basis for evaluation of plant germplasm resources was the phenotype. In this study, 11 quantitative traits in 24 *B. cartharticus* accessions were investigated to estimate breeding potential and phenotypic variation. The descriptive statistics showed variation coefficients ranged from 2.21% to 24.87%, with an average of 11.61%, indicating a high level of phenotypic variance (Table S3). Analysis of UPGMA clusters and PCA showed that all 24 accessions were divided into four groups (A, B, C, and D), which showed a weak relationship with geographical distribution (Fig. 6). Group A contained only one accession (PI 442,077) from Asia, with low biological yield, PH, SD, TN, and wide flag leaf. Group B consisted of four accessions from South America and two from Europe and Asia, and showed relatively high PH, SD, leaf width, and low TN and LFI. Group C contained almost all the accessions from North America (5) and Europe (3), and other accessions from Asia (4) and South America (3), and showed high TN and LFI, and low SD and leaf width. There were two accessions in Group D, PI308506 from South America and PI595118 from North America, which both had a particularly high TN, and low SD with a dwarf phenotype (Fig. 6a, Table S3). Moreover, PI308506 showed narrow leaves and late heading, and PI595118 showed broad leaves and early heading. The PCA analysis also indicated that the first two components accounted for 91.97% of the variance (76.45% and 15.53%, respectively), and that there were four groups (Fig. 6b).

**Fig. 7:**
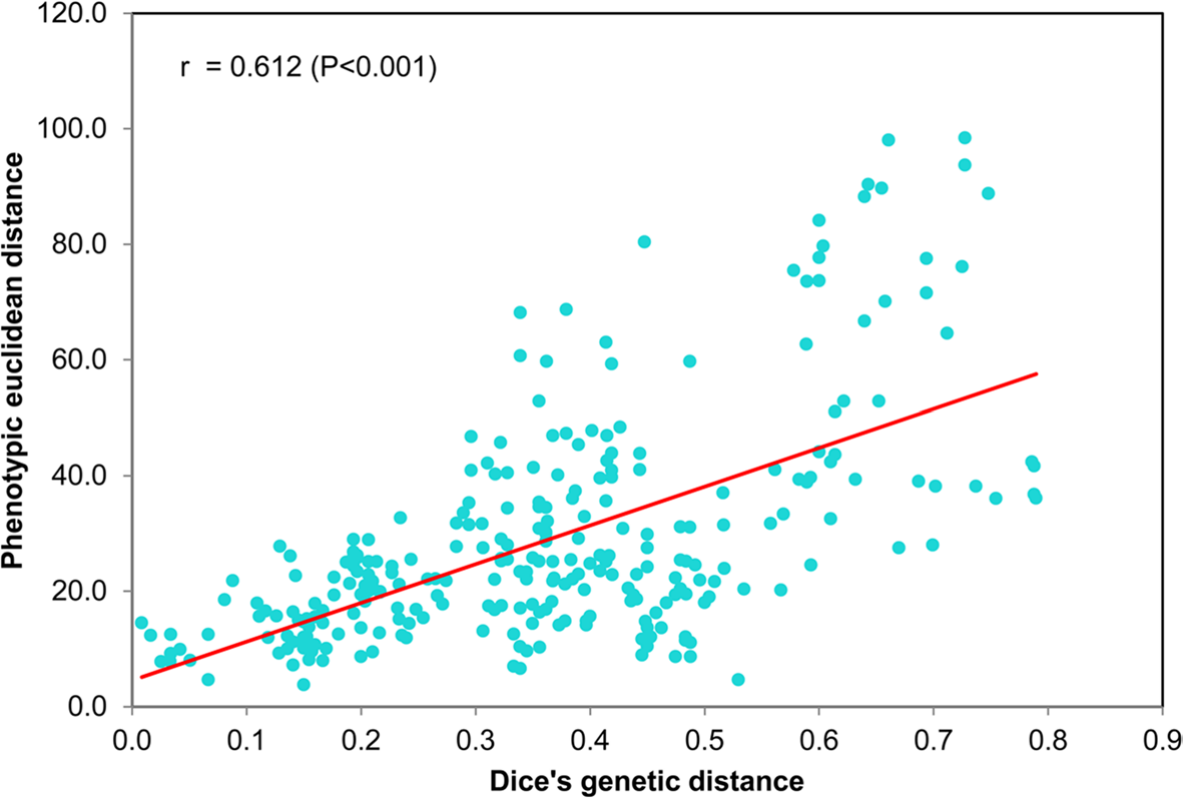
Mantel analysis between genetic distances and phenotypic Euclidean distances of all 24 accessions. The x-axis and y-axis values represent genetic distances and phenotypic Euclidean distances, respectively

Mantel analysis was performed to understand the relationship between genetic distance and 11 traits based phenotypic distance, which showed a high correlation (*r* = 0.612, *P* < 0.01) (Fig. 7). While Mantel analysis was also used to analyze the correlation between the Euclidean distance of 11 phenotypic traits and genetic distance of 24 accessions respectively (Table 5). The results showed that the coefficients ranged from 0.340 to 0.666, and four traits (PH, FLW, SD, and FMY) had high significant correlation (*r* > 0.6, *P* < 0.001). In addition, phenotypic groups were consistent with phylogenetic clusters. The genetic data could mainly explain the phenotypic difference. For example, the low biological yield, PH, SD, TN, and wide flag leaf of PI442077 in Group A might be due to particular genetic information in Cluster IV (Figs. 5, 6 and Table S3). All ten accessions in Cluster I were found in Group C, and all six accessions in Group B were in Cluster II. Both PI308506 and PI595118 in Group D showed dwarf phenotype, with more tillers and thinner stems (Fig. 6, Table S3), but there were significant differences in heading date and leaf width between PI308506 in Cluster IV and PI595118 in Cluster III, which might be explained by a split in genetic clustering.

**Table 5:** Correlation coefficients between Euclidean distances based on 11 traits and genetic distances of 24 accessions

	Euclidean distance										
	DMY	FLL	FLW	HD	LFI	PH	PLL	PLW	SD	TN	FMY
r	0.664	0.412	0.612	0.597	0.369	0.656	0.340	0.532	0.666	0.428	0.664
*P* value	0.010	0.019	0.001	0.003	0.020	0.001	0.038	0.005	0.001	0.007	0.001

Note: r is calculated as correlation coefficient based on the genetic distances of 24 accessions and Euclidean distances of 11 traits

## Discussion

### The transcriptome is a key genetic resource for *Bromus* species

Efforts toward molecular phylogeny and molecular breeding of *Bromus* L. have stagnated due to limited genomic resources. In the present study, our complete transcriptomic analysis in *B. catharticus* covered 11 tissue samples, and likely assembled almost all expressed genes, with a total of 257,773 unigenes, 193,082 (74.9%) of which were annotated. While our data represents a large collection of expressed genes that can be used in future genetic studies and improvement programs in *B. catharticus* and even other *Bromus* species, these numbers are superior to those in some other forage species previously reported, for example *Dactylis glomerata* [19], *Elymus sibiricus* [12] and *Pennisetum purpureum* [20]. It is known that the third-generation sequencing of PacBio and Nanopore could produce up to 1 Mb reads, but the full length of transcripts always prefers to do the pooling of various tissues, to lower the sequencing cost; in addition, their sequence errors are higher than those of Illumina sequencing platforms [21, 22]. Our data may be used as a gene reference in subsequent transcriptomic analyses in *B. catharticus* in the future.

### Tissue-specific gene expression

Grass seeds are the basis of natural grassland restoration and artificial grassland establishment, while stems and leaves are important factors for forage yield and nutritive value [23, 24]. Few tissue-specific transcriptome works on forage grasses have been done to identify candidate genes involved in tissue development and forage quality improvement. Research on elephant grass (*Pennisetum purpureum*) stem lignocellulose identified 3,852 DEGs, and screened 43 candidate genes involved in lignocellulose biosynthesis, which might promote the development of high-quality elephant grass varieties [7]. We found 1,570 stem-specific genes, among which those significantly enriched in the phenylpropanoid biosynthesis pathway might affect the feed value of *B. catharticus*, as phenylpropanoid metabolism is a major pathway of lignocellulose biosynthesis [7, 25]. While we identified 3,030 leaf-specific genes mainly related to the photosynthetic pathways, which might be of benefit in the improvement of leaf photosynthetic efficiency and biomass parameters. Analysis of leaf transcriptome of alfalfa identified 5,133 DEGs and senescence-associated pathways, including ribosome and phenylpropanoid biosynthesis, and starch and sucrose metabolism pathways, and suggested a novel interpretation of the molecular mechanisms of leaf senescence [26]. In addition, our comparative analysis showed that the ribosome pathway was the most significantly enriched pathway among 1,803 seed-specific genes. It is known that ribosomes are needed to synthesize storage and other important proteins, such as late embryogenesis abundant (LEA) proteins with key roles in seed maturation and drying [27], and they are also required in seed germination, where new ribosomes are produced after several hours of imbibition to synthesize proteins related to DNA repair, RNA synthesis, organelle assembly, and energy supply [28, 29]. Simply put, this work provides a preliminary understanding of tissue-specific expression profiling of *B. catharticus*, which may be valuable genomic data for the improvement of the yield and quality of *B. catharticus*.

### Abundant EST-SSRs in *B. catharticus*

EST-SSRs are powerful genetic markers, which have been popularly used to determine genetic diversity, population structure, cultivar identification, genetic maps, and marker-assisted selective breeding due to its advantages in identifying co-dominance, abundant polymorphisms, high transferability, and rapid and convenient detection methods [13, 30]. To date, few EST-SSRs have been applied to *B. catharticus*, and most of the diversity estimation was based on the phenotypic characteristics. Within our transcriptomes from different development and growth stages, with a total of 37,288 SSRs, the occurrence frequency of EST-SSRs was one per 7.8 kb. This frequency was higher than that of alfalfa (1/12.06 kb) [11] and *Leymus chinensis* (1/10.78 kb) [31], but lower than that of *E. sibiricus* (1/6.95 kb and 1/6.2 kb in two previous reports [12, 13]). In addition, the total number of SSRs was far more than that of *E. sibiricus* (8,769 and 8,871 in two reports [12, 13]), *Lolium multiflorum* (11,254) [14], and alfalfa (1,649) [11]. In contrast, the proportion of EST-SSR markers with numbers of high polymorphisms was lower than that of alfalfa (27/100) [11] or *E. sibiricus* (112/500) [12], which may be caused by primer design and selection. The differences in the amount and frequency of SSRs also strongly depends on the species, size of the database, SSR search criteria, and mining tools used. In this study, tri-nucleotide SSR repeats were most abundant, followed by mono- and di-nucleotide repeats among various classes of SSRs, and CCG/CGG was the most in tri-nucleotide repeats, which was identical to those found in *E. sibiricus and L. multiflorum*, but different from the GAA/CTT repeats most abundant in *Sesamum indicum* and *Lupinus luteus*. The abundance of SSRs seems to be species-specific [12, 13, 32, 33]. In brief, the present study identified a large number of molecular markers conducive to breaking the bottleneck problem of genetic research in *B. catharticus*.

### EST-SSRs are valuable tools for germplasm characterization and molecular breeding of *B. catharticus*

Understanding of genetic diversity and population structures is very important for the effective management and utilization of germplasm resources [34]. Evaluation of the genetic variation of forage resources can provide a basis for selection of resources, and thus speed up the progress of high-quality forage breeding. The evaluation of *B. catharticus* germplasms has been relatively lagging, and diversity estimation by molecular markers showing significantly higher amounts and resolution than those of morphological and cellular markers was especially rare [35]. A previous study on the genetic diversity of *B. catharticus* showed that the genetic basis of commercial varieties was very narrow, with an average genetic similarity coefficient of 0.96 [16]. In contrast, the present study contained 24 accessions from four continents, including wild types and uncertain materials, and found an even lower level of genetic similarity (averaged 0.636) than those of commercial varieties. Most accessions from Asia were grouped into one cluster, and 66.67% accessions showed mixed relationships, while 87.50% South America accessions showed a pure origin (Q-value > 80%), which suggested the probability of gene flow among the two regions were different. Based on breeding states, the results suggested higher diversity in wild materials than cultivar and cultivated materials, which might be due to contribution of long-term artificial domestication [36]. The tested wild resources showed higher genetic diversity, which also indicated the possible benefits of breeding comprehensive varieties with wild resources in the future.

Over the long history of evolution, a number of germplasms were formed under genetic and environmental influences, leading to abundant morphological diversity. Phylogenetic analysis of all the accessions were consistent with phenotypic groups. There is a relatively high correlation (*r* = 0.612, *P* < *0.001*) between genetic variation and phenotypic variation, and the phenotypic clusters showed a weak relationship with geographical distribution, suggesting that the phenotypic diversity of *B. catharticus* was mainly due to the contribution of genetic factors. This could be supported by the previous works on *Ceratochloa*, which suggested a high correlation (*r* = 0.70, *p* = 0.001) between morphological variation and DNA polymorphism using AFLP markers [2], and a low correlation (*r* =  − 0.243) between geographical distance and genetic similarities among hexaploid *Bromus* accessions [3]. Phenotypic diversity analysis in Argentinian populations suggested total phenotypic variation of *B. catharticus* was mostly due to the environmental component [37]. The inconsistency between this study and others might be caused by different sources of tested germplasms and collection of phenotypic traits. In this study, we also suggested FLW, PH, SD, and FMY might be more reliable and effective traits in a selective breeding project; in particular, SD as an indicator of major biomass traits showed relatively high variation coefficients and high correlation with molecular data, which could be highly effective and stable in marker-trait association or QTL mapping. The identified EST-SSR markers may potentially be used for further genetic selection and marker-assisted breeding.

## Conclusions

In this study, in *B. catharticus*, we conducted RNA-seq analysis to identify 1803, 3030, and 1570 genes specifically and highly expressed in seed, leaf, and stem, respectively. We also generated 257,773 unigenes based on a total of 11 RNA-seq datasets, which were used to develop 37,288 EST-SSRs. And 52 polymorphic EST-SSR markers were selected to analyze genetic diversity among 24 *B. catharticus* accessions. A low genetic diversity was found in all the accessions, albeit with higher ones in South America and Asia and wild accessions. The phylogenetic analysis clustered all the accessions into four clusters, which were consistent with the phenotypic clustering results. Furthermore, Mantel analysis suggested the total phenotypic variation was mostly contributed by genetic component, and four phenotypic parameters, including stem diameter, plant height, leaf width, and biomass yield, were significantly correlated with genetic variations, suggesting a great potential in genetic selection and breeding. In general, a comprehensive genomic resource was generated that could benefit genetic and taxonomic studies, as well as molecular breeding for *B. catharticus* and its relatives in the future.

## Methods

### RNA isolation and transcriptome sequencing

**Fig. 8:**
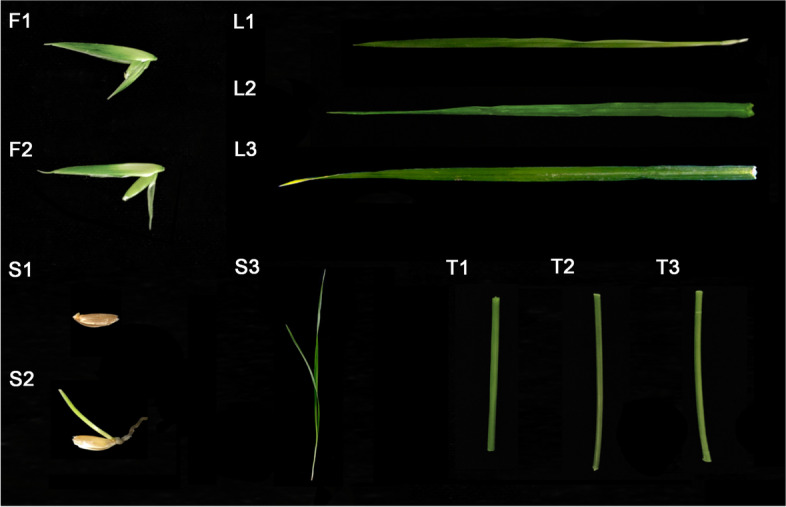
Tissue samples used in this study. A total of eleven samples were collected for RNA-seq, including seeds emerging with a radicle length of 1–2 mm (S1) and shoot length of 1 cm (S2), aboveground parts of seedlings at the three-leaf stage (S3), leaves at the tillering stage (L1), flag leaves (L2) and penultimate leaves (L3) at 8 DAF, stems at tillering stage (T1), 8 DAF (T2), and 20 DAF (T3), and seeds at 8 DAF (F1) and 20 DAF (F2). All samples were collected and mixed from at least ten individual plants

BCS1103, a new line of *B. catharticus* with an excellent yield, was originally selected via wild germplasm collected in the southwest region of Sichuan Province, China. The single plant was grown to maturity in a pot with recommended cultural practices at Sichuan Agricultural University, Yaan, Sichuan. Eleven tissue samples of this accession were collected from at least ten healthy individuals, i.e., seeds with emerged radicle length of 1–2 mm (S1) and with shoot length of 1 cm (S2), aboveground seedlings at the three-leaf stage (S3), leaves at tillering stage (L1), flag leaves (L2), and penultimate leaves (L3) at 8 days after fertilization (DAF); stems at tillering stage (T1) at 8 DAF (T2) and 20 DAF (T3); and seeds at 8 DAF (F1) and 20 DAF (F2) (Fig. 8). All tissues were placed directly into liquid nitrogen before being stored at − 80 °C. Total RNA was isolated using the method described by Ghawana et al. [38]. A total amount of 1.5 μg RNA per sample was used for library construction. Sequencing libraries were generated using NEBNext® Ultra™ RNA Library Prep Kit for Illumina® (NEB, USA) following the manufacturer’s recommendations, and index codes were added to attribute sequences to each sample. The libraries were sequenced on an Illumina Hiseq 2500 platform.

### *De novo* assembly and gene annotation

In this study, clean reads were obtained by removing those reads that contained adapters or poly-N (i.e., ambiguous sequences), and low-quality reads that had more than 50% of bases with Q-value ≤ 20. The unigenes were binned by Corset [39] from the assembled contigs using Trinity [40]. All unigenes were annotated against seven databases: the NCBI non-redundant protein database (Nr), the NCBI nucleotide database (Nt), Pfam, Swiss-Prot, Cluster of Orthologous Group, Gene Ontology (GO), and Kyoto Encyclopedia of Genes and Genomes (KEGG). NCBI Nt and Nr alignments were considered superior annotations if there were inconsistent results among these databases.

### Gene expression and DEGs identification

RSEM software was used for mapping clean reads onto the unigenes [41]. Gene expression levels were estimated based on FPKM (Fragments Per Kilobase of exon model per Million mapped fragments) values. The expression patterns of each sample reflected by the density distribution of FPKM values were plotted based on a log_10_ (FPKM) scale as fold change. Moreover, tissue-specific genes were identified by overlapping upregulated DEGs in the two comparisons using DEGseq with a threshold set of adjusted *p*-value < 0.05 and |log_2_ fold change|> 1 [42].

### Quantitative real-time PCR (qRT-PCR) analysis

To experimentally validate our results, eleven genes were selected for qRT-PCR analysis on the three samples (F2 for seed, L2 for leaf and T2 for stem), with three biological replicates. Details of the eleven genes and their primer pairs were shown in Table S4. The first-strand cDNA was synthesized according to Revert Aid Premium Reverse Transcriptase Kit (Thermo Scientific™). The experiment was performed in a CFX96 Real-Time System (Bio-Rad, USA) with *GAPDH* (glyceraldehyde-3-phosphate dehydrogenase) gene as internal control. Each PCR reaction contained 10 μl of the SYBR-Green Master Mix and 4 pmol of each primer, and the amplification was set under the following condition: an initial step of 95 °C for 3 min and a three-step cycle of 95 °C for 10 s, 57 °C for 10 s and 72 °C for 15 s, repeated 40 times. Gene quantification was determined using the ΔΔCT method. The relative expression values normalized using log_2_(fold change) were used to calculate the correlation coefficient between RNA-seq and qRT-PCR results.

### EST-SSR detection and primer design

MISA software was used to detect potential SSR markers in the unigenes [43]. Default parameters were set to include mono-nucleotide repeats of more than 10X, di-nucleotide repeats of more than 6X, and tri-, tetra-, penta-, and hexa-nucleotide repeats of more than 5X. Primer 3 was used to design PCR primers for each SSR with the default parameters [44]. PCR primers were synthesized in TsingKe Biological Technology Company (Beijing, China).

### SSRs validation and genetic variability

A total of 24 accessions were collected from ten countries across four continents (North America, South America, Europe, and Asia), including wild materials, cultivars, and accessions of uncertain breeding status (Table 6). Seeds of BCS1103 and Jiangxia were provided by Sichuan Agricultural University, and the others were obtained from the National Plant Germplasm System (USA). Seeds were planted in basins until three-leaf stage, in a randomized complete block design with three replicates at Sichuan Agricultural University (Ya’an, Sichuan, China) during the crop season in 2016 and 2017. In each plot, intra-row spacing of 0.5 m and inter-row spacing of 0.5 m allowed four plants per row, twelve plants in total. The bulked samples were mixed with equal amounts (50 mg) of fresh leaf tissues of 20 individuals per accession, and subjected to DNA extraction using a plant DNA extraction kit (Tiangen, Beijing, China) following the manufacturer’s instructions. PCR amplification was performed in a reaction volume of 15 μl, containing 1.5 ng template DNA, 1 U of Taq polymerase, 6 μM reverse and forward primers, and 7.5 μM Mix solution. The PCR reaction cycling profile was initially 95 °C for 10 min, followed by 35 cycles of 50 s at 95 °C, 50 s at the annealing temperature, and 1 min at 72 °C, with a final extension for 10 min at 72 °C. The EST-SSR PCR products were loaded into 6% urea polyacrylamide gels in 1X TBE buffer for electrophoresis at 200 V for 2 h, based on the size of amplified DNA fragments. Afterwards, the gel was subjected to 2–3 initial washing steps prior to staining with 0.1% silver nitrate for 15 min. The gel was immediately washed and fixed in developer solution for visualization of nucleotide bands.

**Table 6:** Accessions of *B. catharticus* used in the study

No.	Accession code	Origin	Breeding status	Continents
1	PI331171	Argentina	Uncertain	South America
2	PI331172	Argentina	Uncertain	South America
3	PI197848	Argentina	Uncertain	South America
4	PI309955	Rio Grande do Sul, Brazil	Uncertain	South America
5	PI309957	Rio Grande do Sul, Brazil	Uncertain	South America
6	PI309958	Rio Grande do Sul, Brazil	Uncertain	South America
7	PI308506	Peru	Wild material	South America
8	PI204157	Uruguay	Wild material	South America
9	PI495807	Inner Mongolia,Ongniud Qi, China	Cultivar	Asia
10	Jiangxia	Hubei, China	Cultivar	Asia
11	BCS1103	Sichuan, China	Cultivar	Asia
12	PI217593	Ootacamund, India	Wild material	Asia
13	PI377533	Tokyo, Japan	Wild material	Asia
14	PI442077	Kanagawa, Japan	Cultivar	Asia
15	PI413792	France	Uncertain	Europe
16	PI618750	France	Cultivated material	Europe
17	PI634281	France	Cultivar	Europe
18	PI292258	England, United Kingdom	Uncertain	Europe
19	PI187000	Alaska, United States	Wild material	North America
20	PI409139	Maryland, United States	Wild material	North America
21	PI595114	Alabama, United States	Wild material	North America
22	PI595115	Alabama, United States	Wild material	North America
23	PI595118	Mississippi, United States	Wild material	North America
24	W610415	Louisiana, United States	Wild material	North America

Polymorphic information content (PIC), marker index (MI), and resolving power (Rp) were calculated to assess the amplification efficiency of SSRs. PIC was calculated for each primer pair according to the formula: PIC_i_ = 2 *f*_*i*_ (1 − *f*_*i*_), where PIC is the polymorphic information content of marker i, *f*_*i*_ is the frequency of the fragments, which were present, and “1 − *f*_*i*_” is the frequency of the fragments, which were absent. PIC was averaged over the fragments for each primer pair. MI was calculated following the formula from Powell et al. [45]: MI = PIC × EMR, where EMR (effective multiple ratio, EMR = PP × NPF) is defined as the product of the percent of polymorphic loci (PP) and the number of polymorphic fragments (NPF). The Rp of each primer pair was calculated according to the formula of Prevost and Wilkinson [46]: Rp = ∑Ib, where Ib is the fragment informativeness, calculated as: Ib = 1–[2 ×|0.5 − p|], where p is the proportion of the accessions containing the fragment. Genetic diversity was measured by the number of different alleles (Na), number of effective alleles (Ne), Shannon's information index (I), and expected heterozygosity (He), which were calculated using GENALEX version 6.5 [47].

Pair-wise (Dice) genetic distance was calculated, and the matrix was used to construct a cluster using the Unweighted Pair Group Method with Arithmetic (UPGMA) in Free Tree software with 1000 bootstrap repetitions [48]. Moreover, a Bayesian model-based clustering method was used to cluster accessions into subpopulations using STRUCTURE software (version 2.3.4) [49], and K-means clustering was used to identify the most likely number of genetic groups (K) by calculating ΔK [50]. Structure simulations were carried out using an admixed model, and based on initials trails, the membership of each genotype was tested for the range of genetic clusters from K = 2 to K = 10 (each with 10 independent runs). For each value of K, the burn-in period was set to 50,000 iterations, and estimations were performed for 100,000 Markov Chain Monte Carlo replicates in each run. CLUMPP [51] was used with the Greedy Algorithm method, and the graphical display (barplot) of population structure was generated using DISTRUCT [52].

### Phenotypic and Mantel analysis

Phenotypic data was collected at the full-flowering stage in April, 2017, except for biomass yield and heading date. Nine individual plants were selected randomly from three replicate plots (three per plot) for observations of 11 morphological traits: plant height (PH, cm), stem diameter (SD, mm), flag leaf length (FLL, mm), flag leaf width (FLW, mm), penultimate leaf length (PLL, cm), penultimate leaf width (PLW, mm), length of first internode (LFI, cm), tiller number (TN), days from seeding to heading (DSH, days), dry matter yield (DMY, g/plant), and fresh matter yield (FMY, g/plant) [37] (see measuring methods in Table S5). Data analysis was performed using NTSYS (version 2.2) and PAST (version 3.02) software [53, 54], including descriptive statistics, PCA, and UPGMA clustering. Finally, a Euclidean distance matrix of morphological traits was calculated using NTSYS, and the genetic distance and phenotypic distance matrix were used for Mantel analysis through GENALEX version 6.5 [47].

## Supplementary Information

**Additional file 1: Figure S1.** Length distribution of unigenes and transcripts.

**Additional file 2: Figure S2.** Gene expression patterns. (a) Density distributions of FPKM based on log10 (FPKM) showed the gene expression level over tissues and developmental stages. (b) FPKM interval distribution under different tissues were detected over all levels.

**Additional file 3: Figure S3.** Annotated unigenes of *B. catharticus*. (a) GO categories. The x-axis indicates subcategories within each GO category, and the y-axis indicates the genes number of a specific category; (b) KEGG classification. The x-axis indicates the percentage of genes assigned to a specific pathway, and y-axis indicates the KEGG pathways, including cellular processes (A), environmental information processing (B), genetic information processing (C), metabolism (D) and organismal systems (E).

**Additional file 4: Figure S4.** Validation of EST-SSRs amplified by ESP-178 and ESP-372 primer pairs. The two gel images were cropped to composite a combined graph which could clearly show the EST-SSR validation results, and the original and full-length gel images were provided in Additional file 5.

**Additional file 5: Figure S5.** The original and full-length gel images for amplification products of part polymorphic primers pairs on studied accessions. The accessions numbers showed in Table 6 were labeled on each gel image, and the bands surrounded by red frames from ESP-178 and ESP-372 were verified by sequencing the amplified products. The markers were on both sides of each gel image.

**Additional file 6: Table S1.** Repeat distribution of mono-, di-, tri-, quard-, penta- and hexa- nucleotides.

**Additional file 7: Table S2.** Details of 52 relevant EST-SSR markers.

**Additional file 8: Table S3.** Descriptive statistics of 11 traits for 24 *B. catharticus* accessions.

**Additional file 9: Table S4.** Details of eleven genes and their primers for qRT-PCR analysis.

**Additional file 10: Table S5.** Morphological trait list of *B. catharticus* : names, abbreviations and measuring methods.

## Data Availability

Raw sequencing data used in the current study are available in National Center for Biotechnology Information (NCBI) Sequence Read Archive database under the BioProject PRJNA670326 (https://www.ncbi.nlm.nih.gov). Other datasets supporting the conclusions of this article are included within the article and its additional files. Any reasonable requests are available from the corresponding author.

## Abbreviations

AFLP: Amplified fragment length polymorphism
COG: Cluster of Orthologous Group
DEG: Differentially expressed gene
EST-SSR: Expressed sequence tag-simple sequence repeat
FPKM: Fragments per kilobase per million
GO: Gene ontology
KEGG: Kyoto encyclopedia of genes and genomes
MCMC: Markov Chain Monte Carlo
NGS: Next generation sequencing
PCA: Principal component analysis
RAPD: Random amplified polymorphic
UPGMA: Unweighted pair group method with arithmetic
