# Supplementary figures and images for: Transcriptomic resources for prairie grass (Bromus catharticus): expressed transcripts, tissue-specific genes, and identification and validation of EST-SSR markers
Source: BMC Plant Biol. 2021 Jun 7;21:264. doi: 10.1186/s12870-021-03037-y (PMC8186225; doi:10.1186/s12870-021-03037-y)

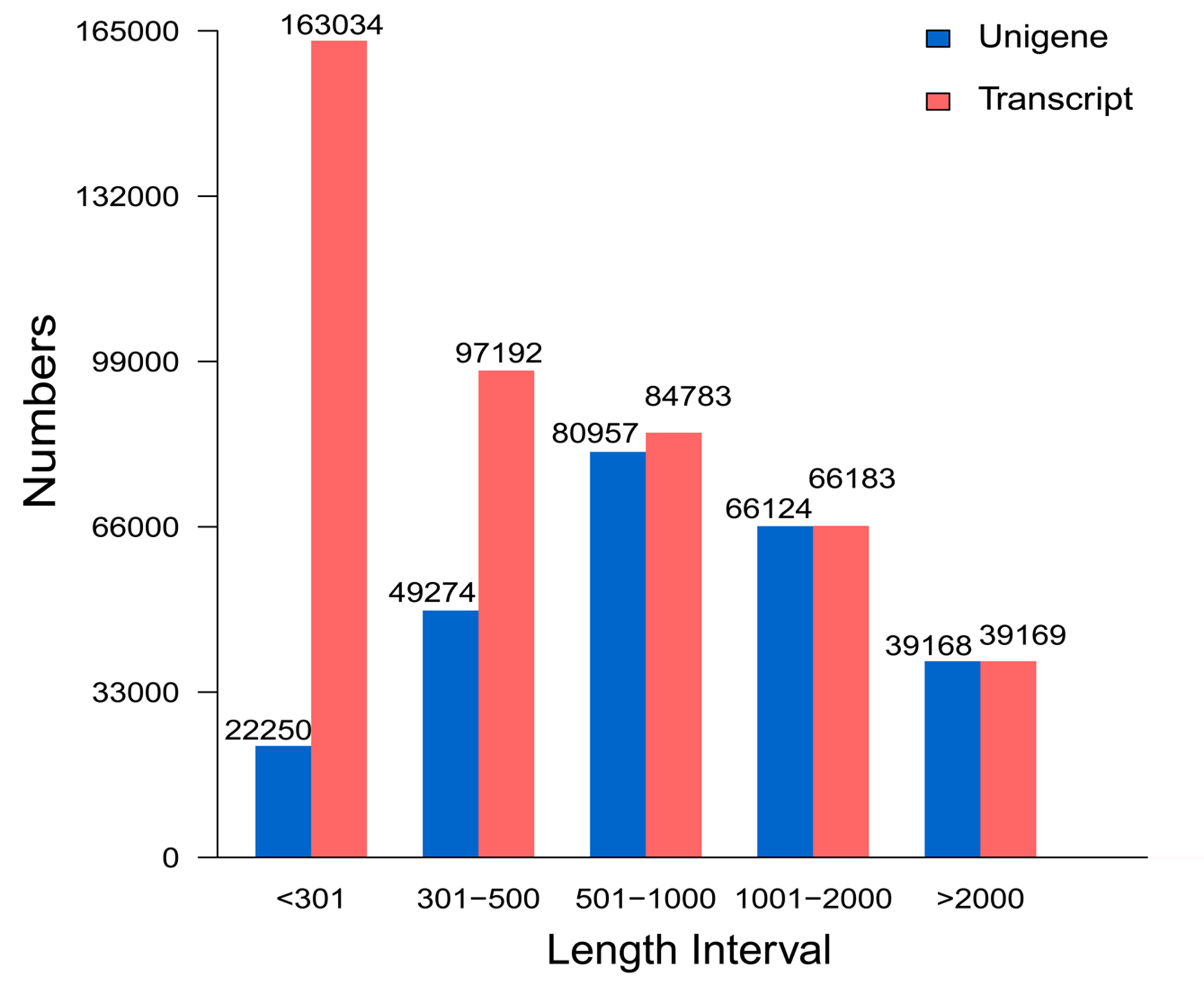

Supplement: Supplementary file 1 — Additional file 1: Figure S1. Length distribution of unigenes and transcripts. [file 12870_2021_3037_MOESM1_ESM.tif]

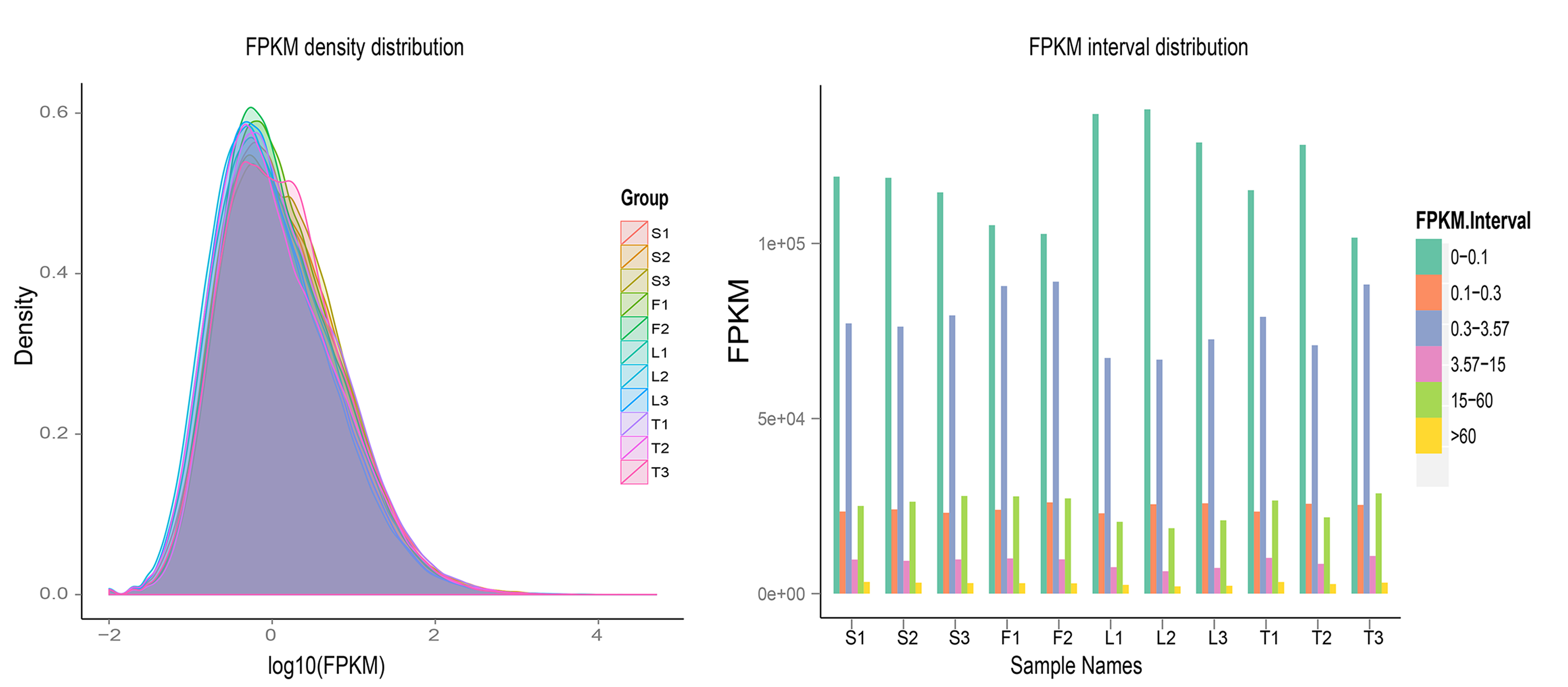

Supplement: Supplementary file 2 — Additional file 2: Figure S2. Gene expression patterns. (a) Density distributions of FPKM based on log10 (FPKM) showed the gene expression level over tissues and developmental stages. (b) FPKM interval distribution under different tissues were detected over all levels. [file 12870_2021_3037_MOESM2_ESM.tif]

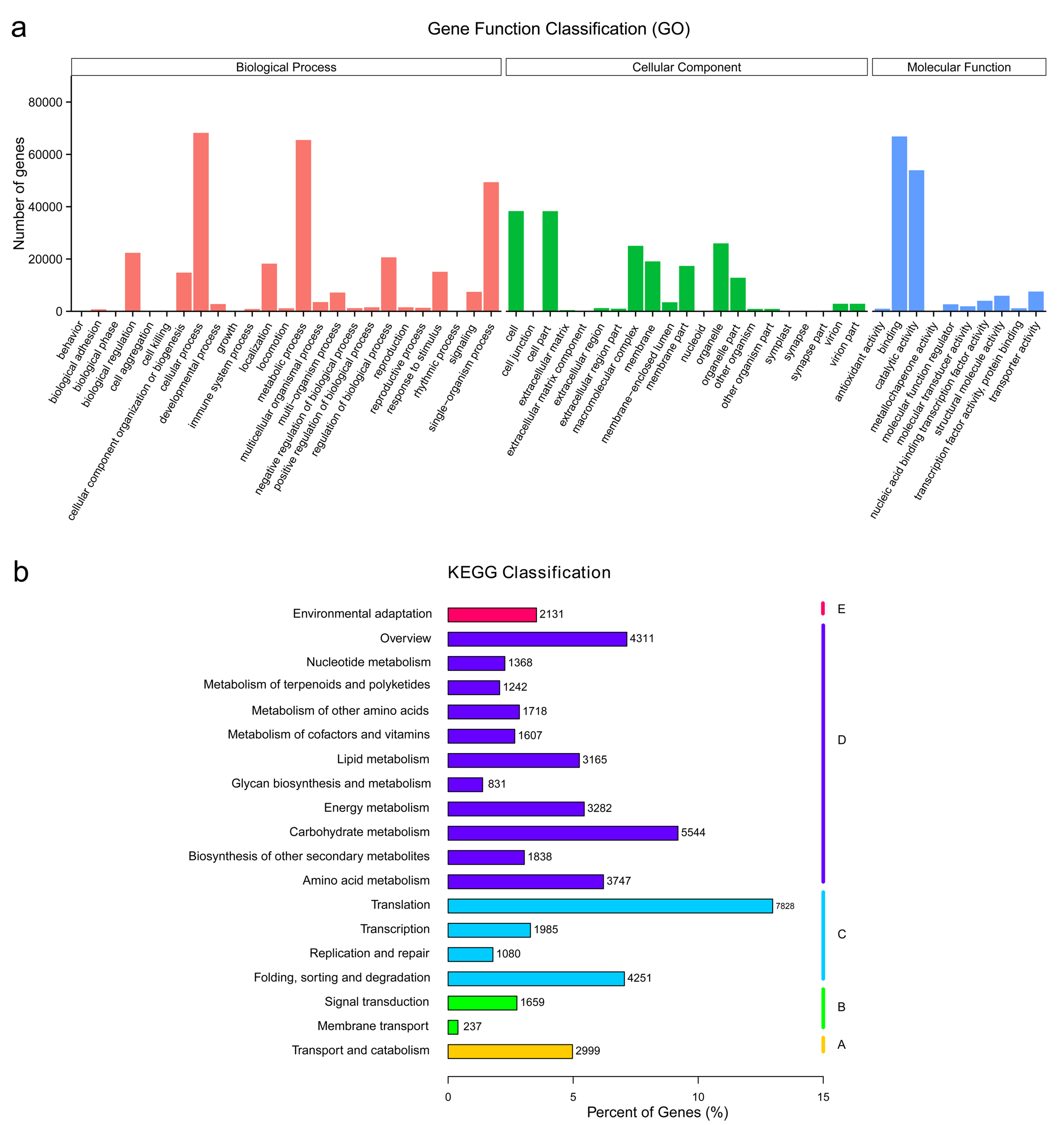

Supplement: Supplementary file 3 — Additional file 3: Figure S3. Annotated unigenes of B. catharticus. (a) GO categories. The x-axis indicates subcategories within each GO category, and the y-axis indicates the genes number of a specific category; (b) KEGG classification. The x-axis indicates the percentage of genes assigned to a specific pathway, and y-axis indicates the KEGG pathways, including cellular processes (A), environmental information processing (B), genetic information processing (C), metabolism (D) and organismal systems (E). [file 12870_2021_3037_MOESM3_ESM.tif]

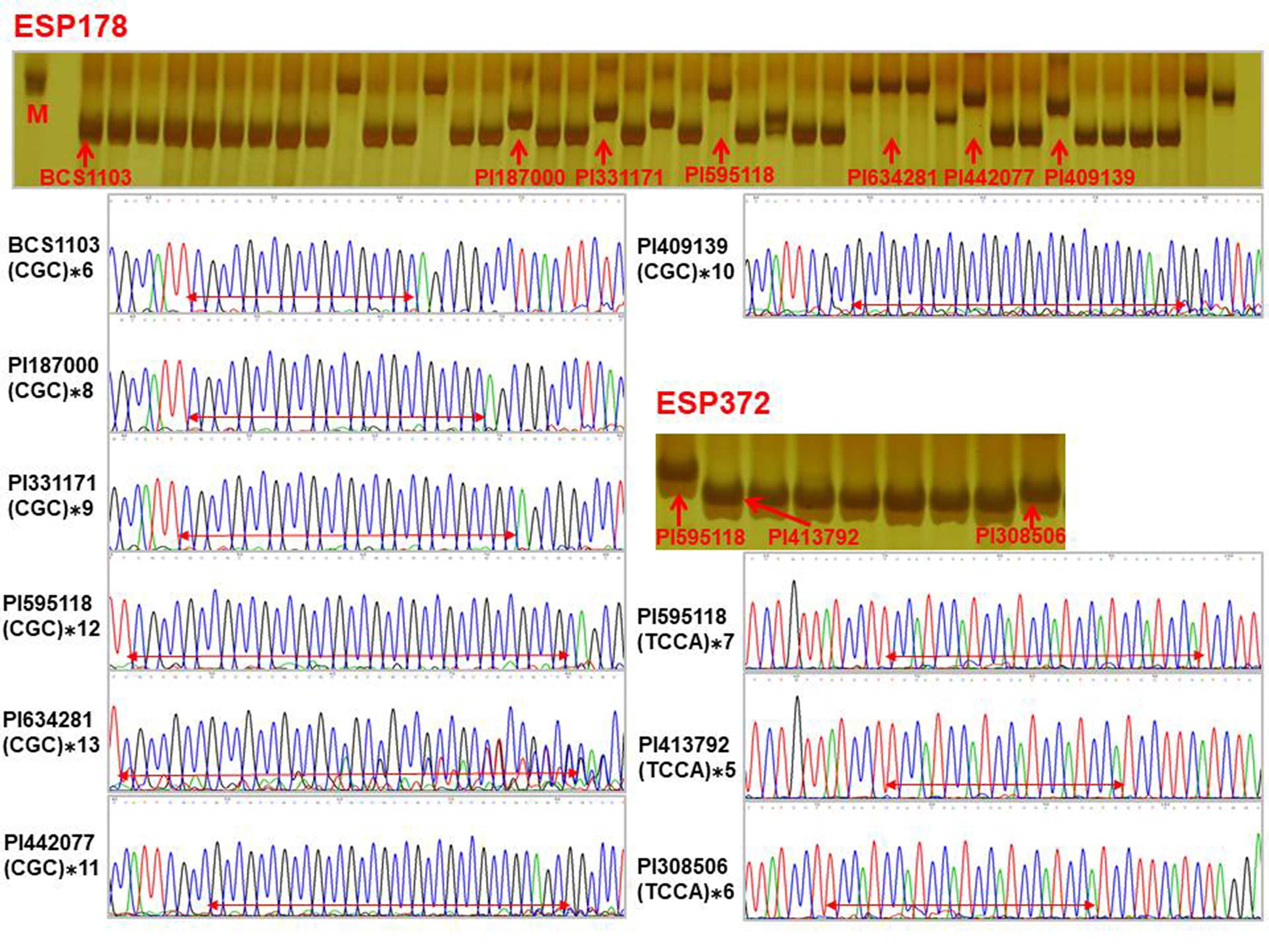

Supplement: Supplementary file 4 — Additional file 4: Figure S4. Validation of EST-SSRs amplified by ESP-178 and ESP-372 primer pairs. The two gel images were cropped to composite a combined graph which could clearly show the EST-SSR validation results, and the original and full-length gel images were provided in Additional file 5. [file 12870_2021_3037_MOESM4_ESM.tif]
