# Supplementary material for: Transcriptomic resources for prairie grass (Bromus catharticus): expressed transcripts, tissue-specific genes, and identification and validation of EST-SSR markers
Source: BMC Plant Biol. 2021 Jun 7;21:264. doi: 10.1186/s12870-021-03037-y (PMC8186225; doi:10.1186/s12870-021-03037-y)
Supplement: Supplementary file 5 — Additional file 5: Figure S5. The original and full-length gel images for amplification products of part polymorphic primers pairs on studied accessions. The accessions numbers showed in Table 6 were labeled on each gel image, and the bands surrounded by red frames from ESP-178 and ESP-372 were verified by sequencing the amplified products. The markers were on both sides of each gel image. [file 12870_2021_3037_MOESM5_ESM.pdf]

ESP-27

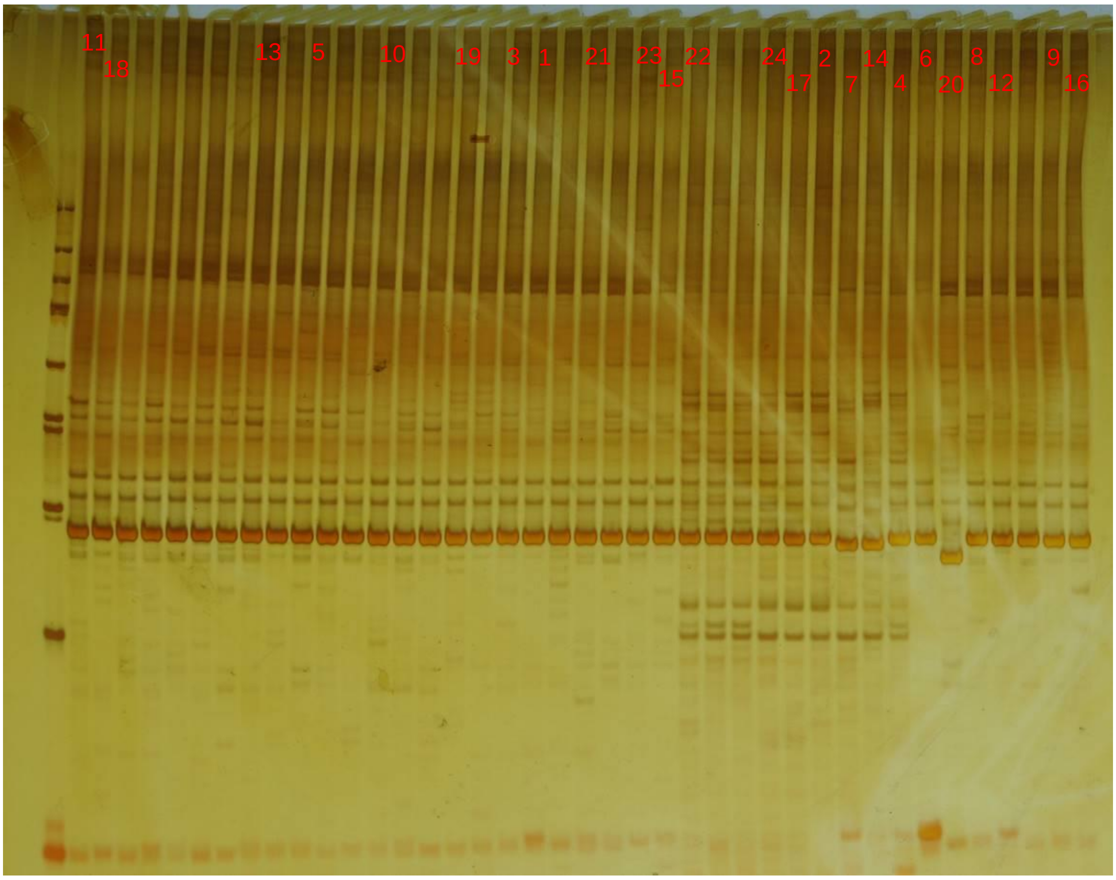

ESP-151

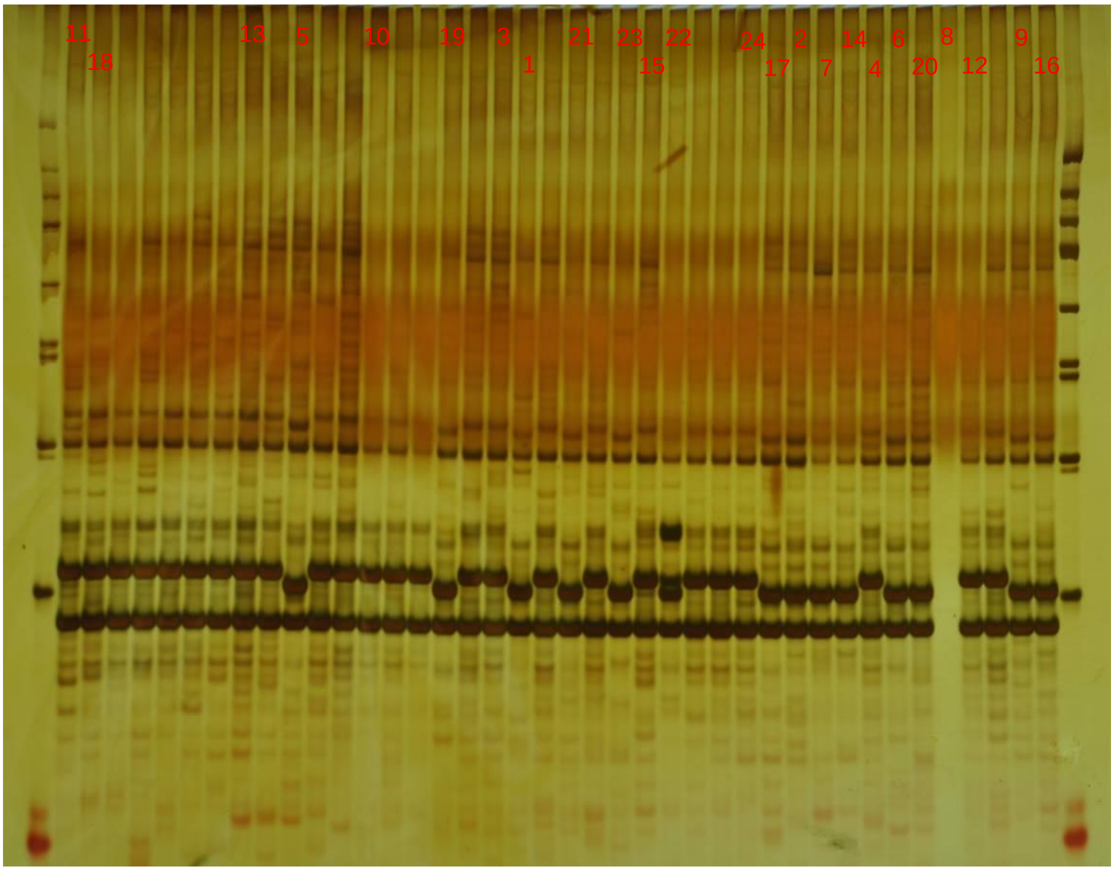

ESP-178

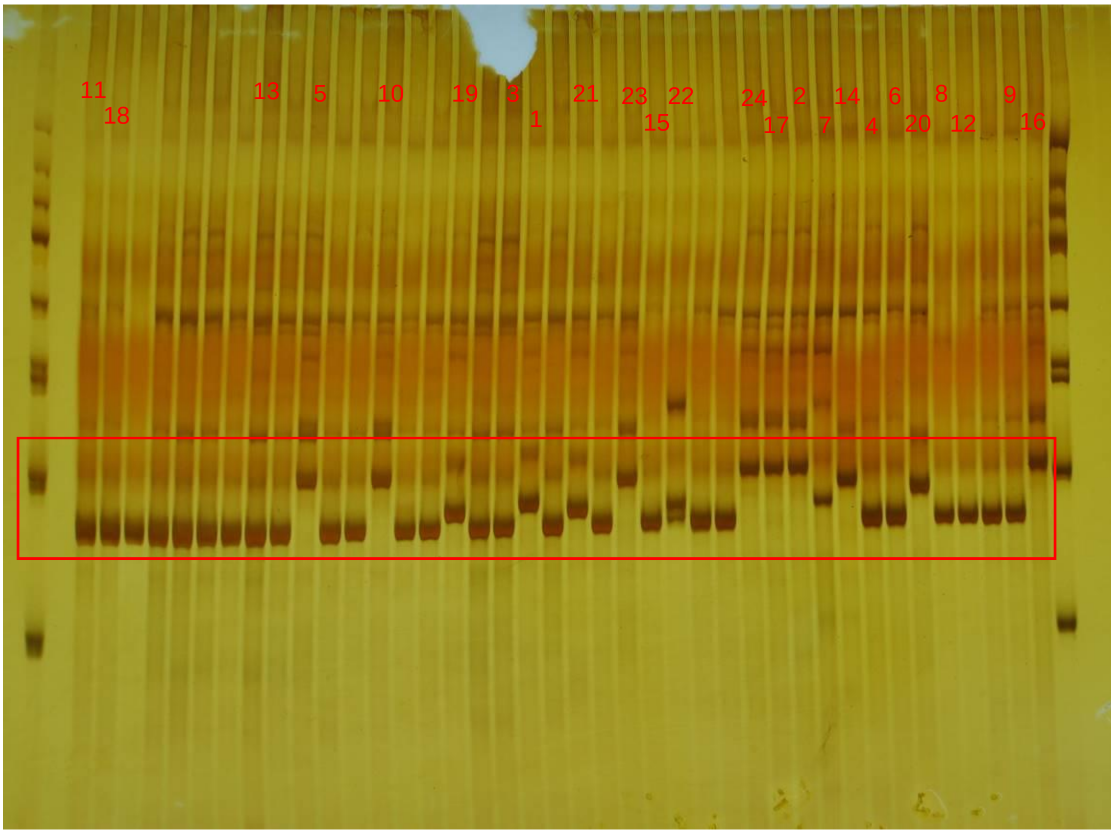

ESP-182

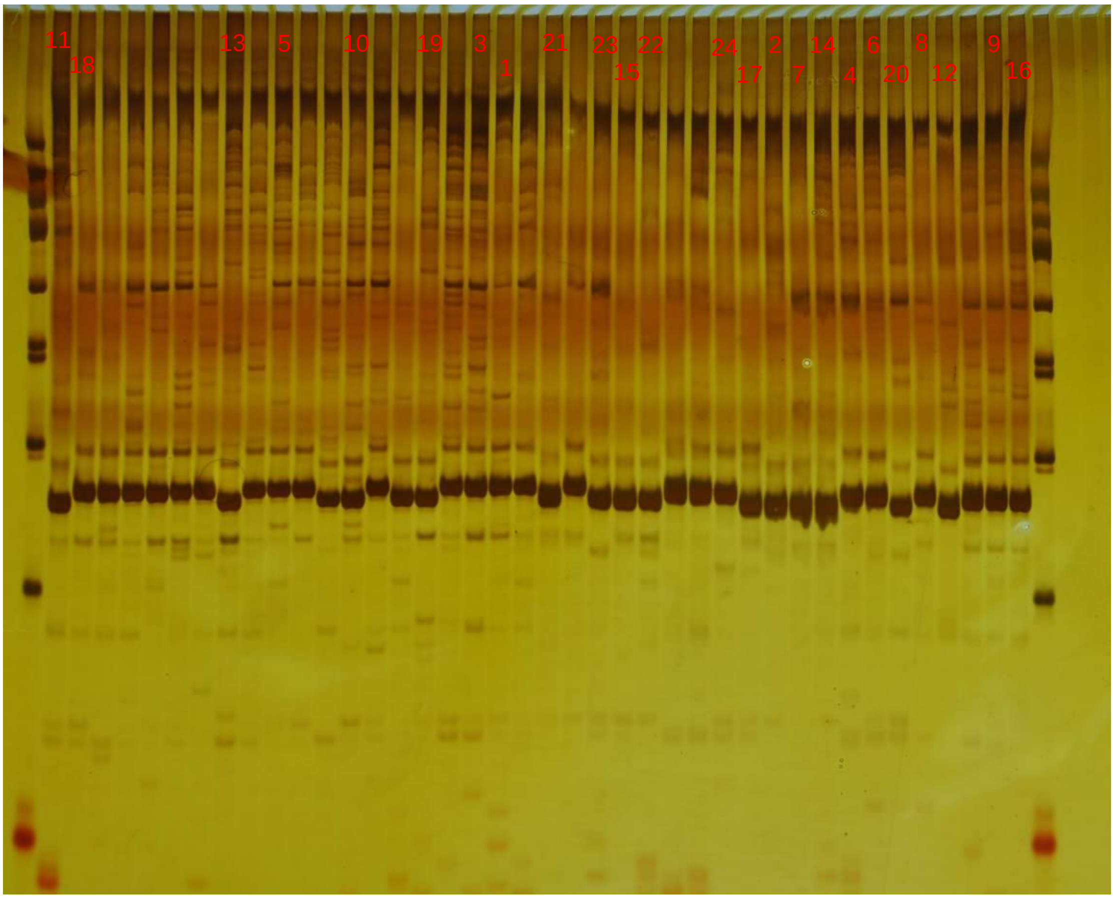

ESP-193

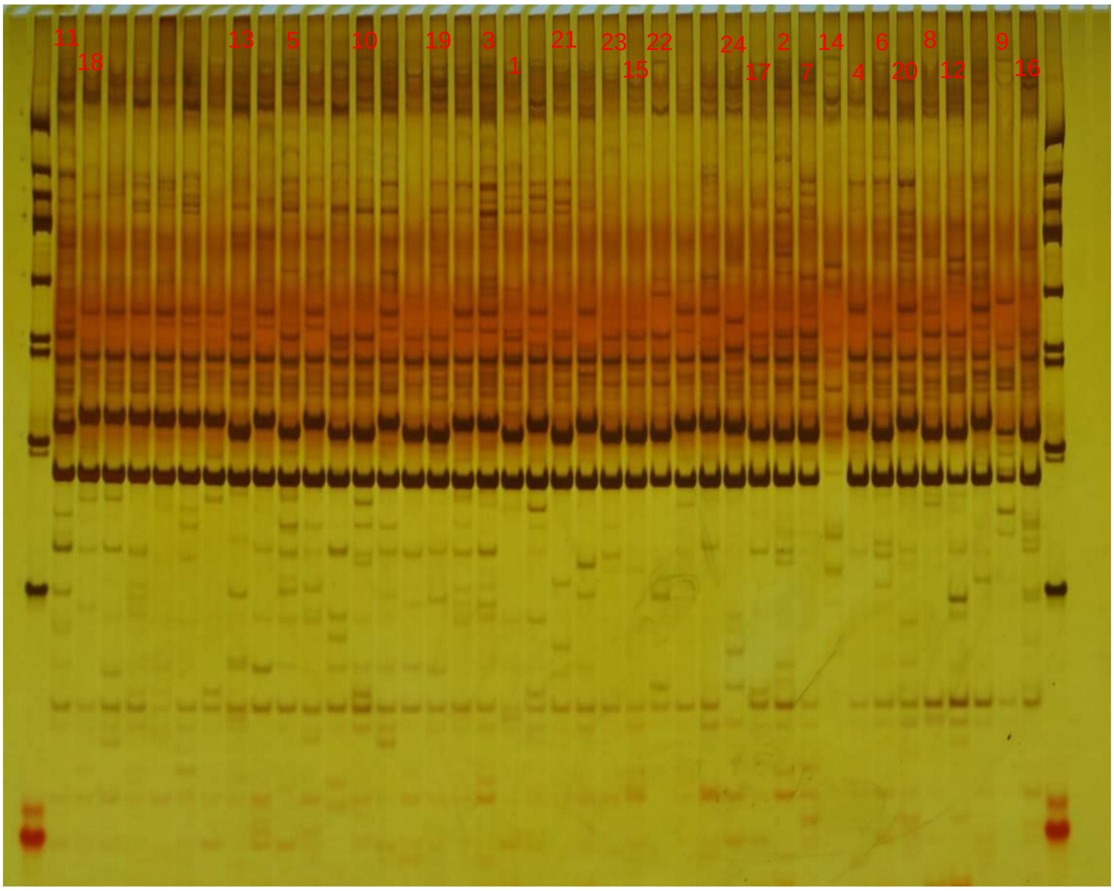

ESP-215

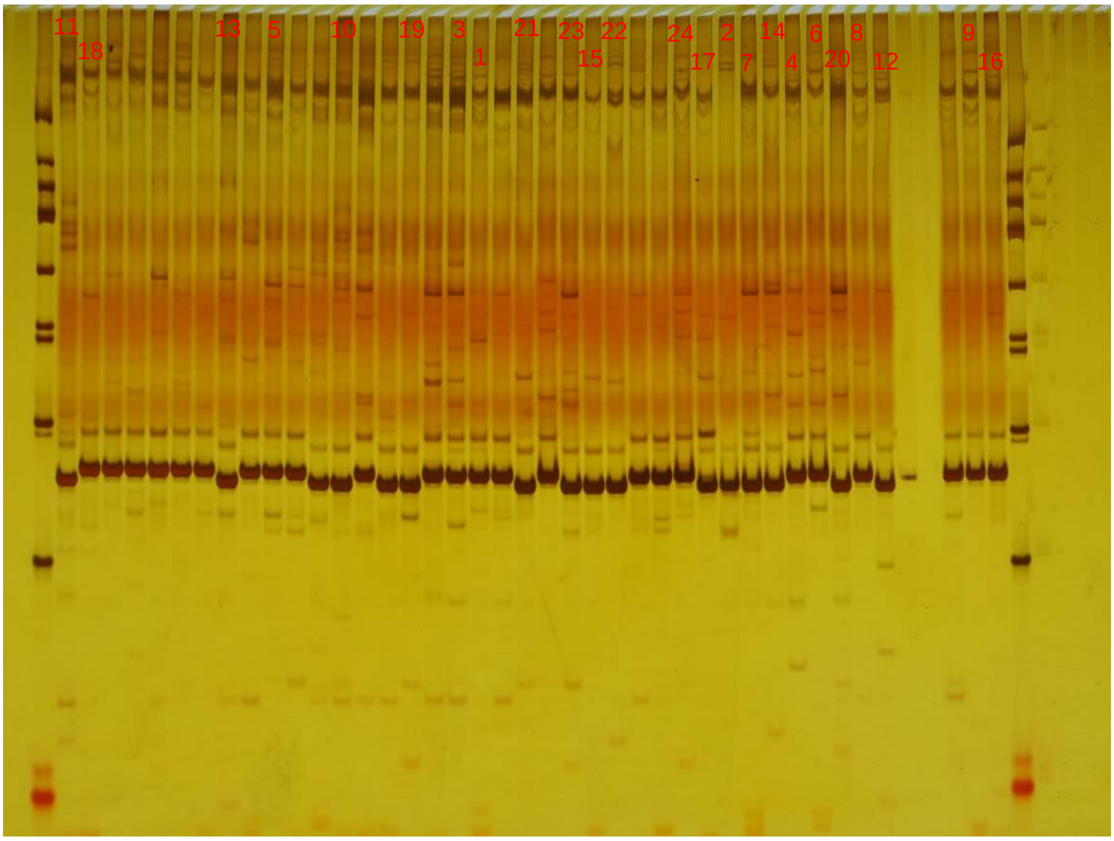

ESP-251

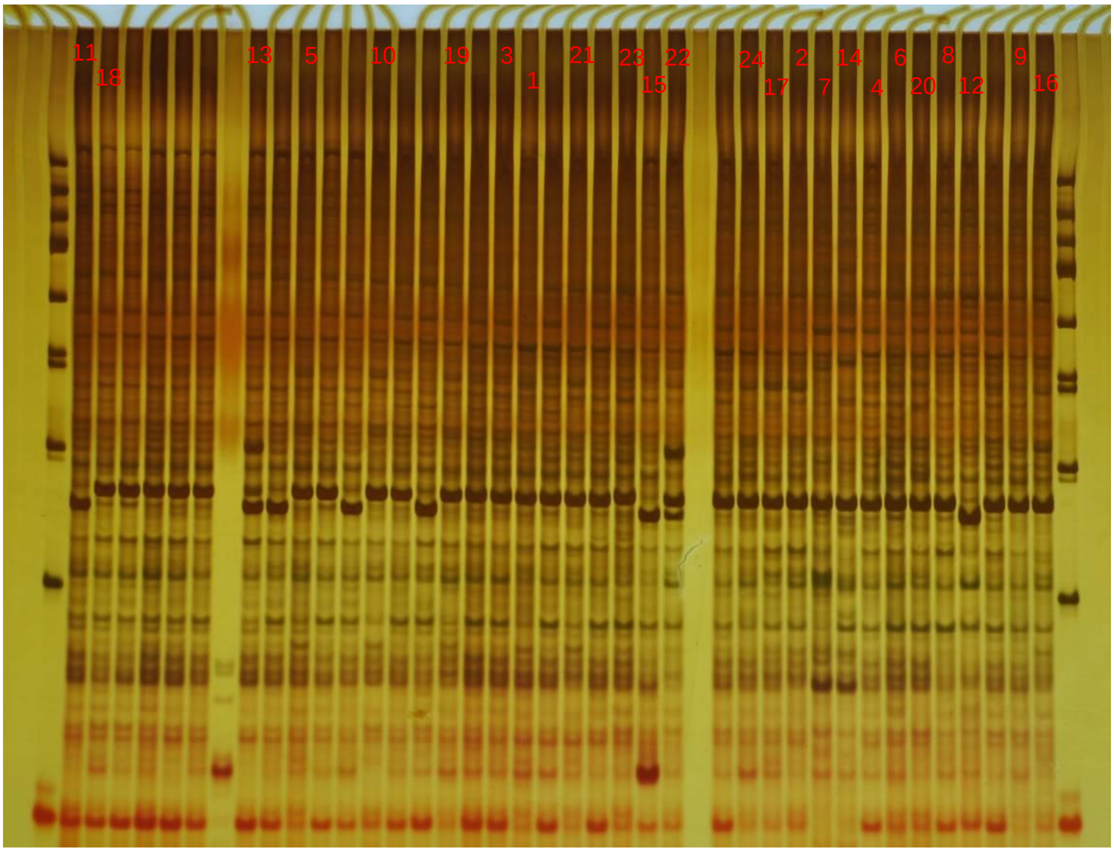

ESP-289

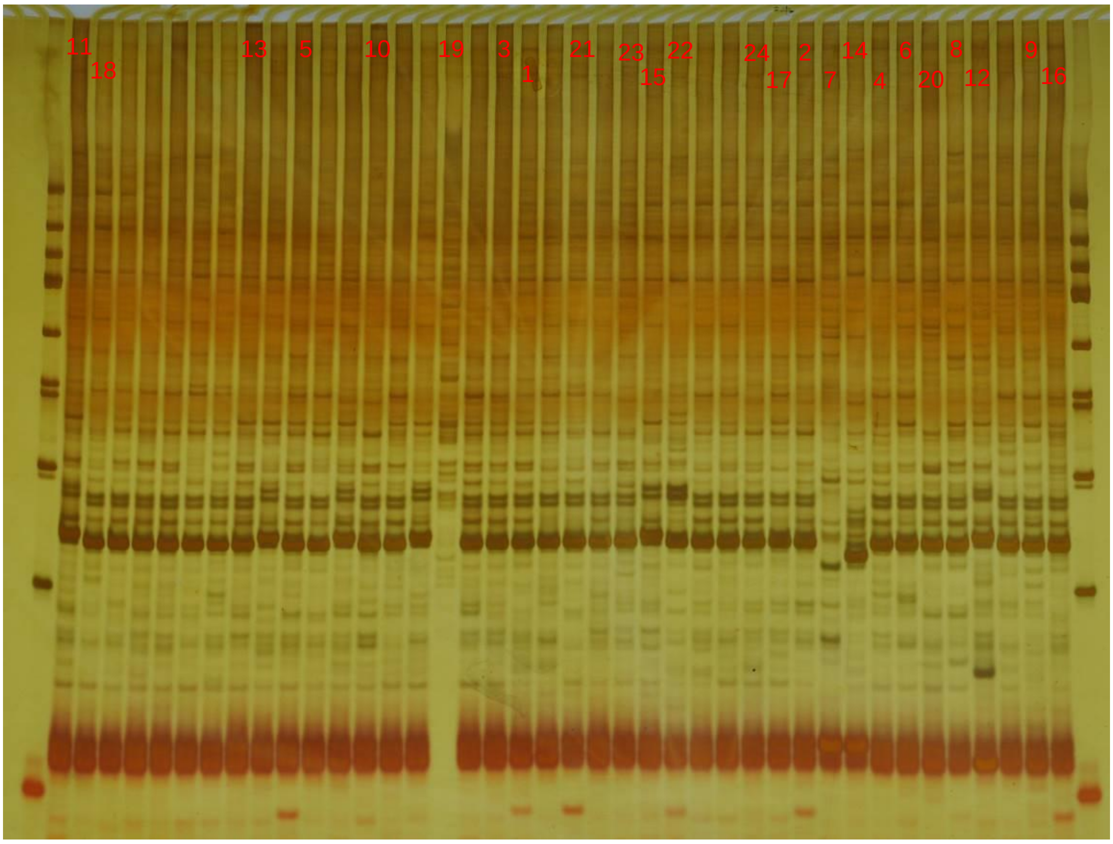

ESP-302

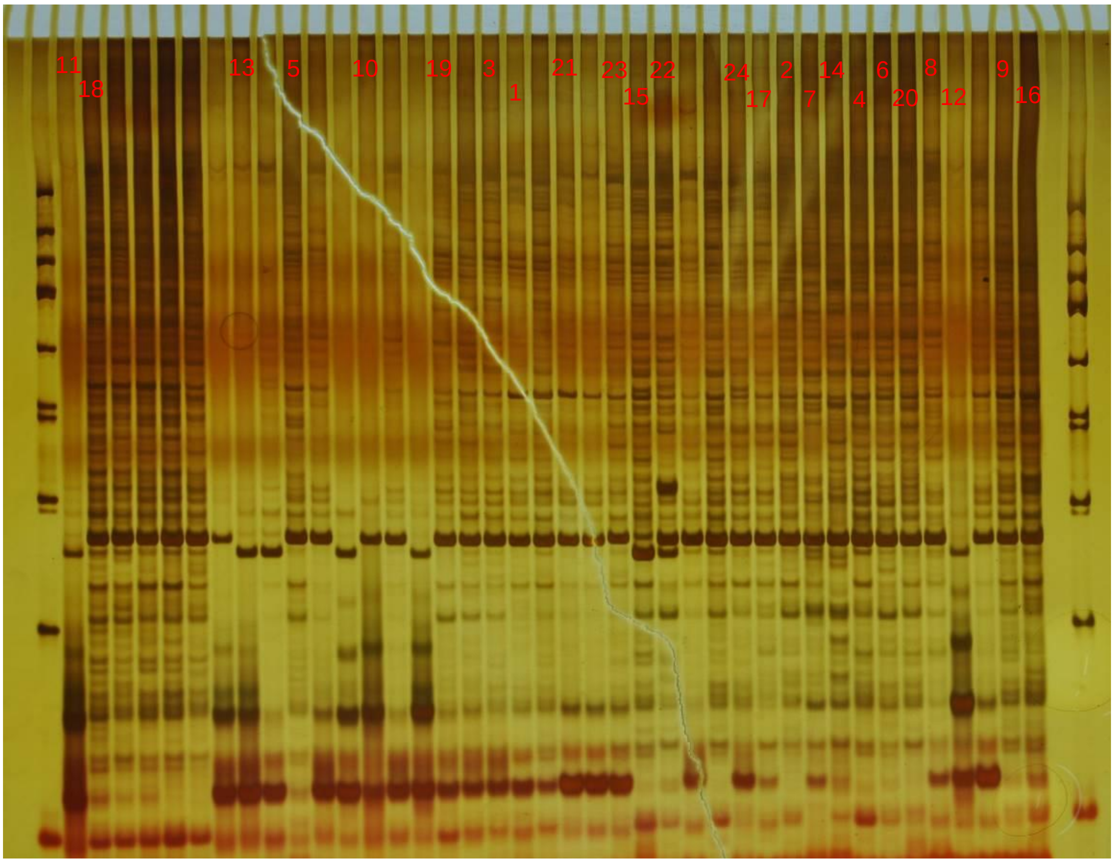

ESP-319

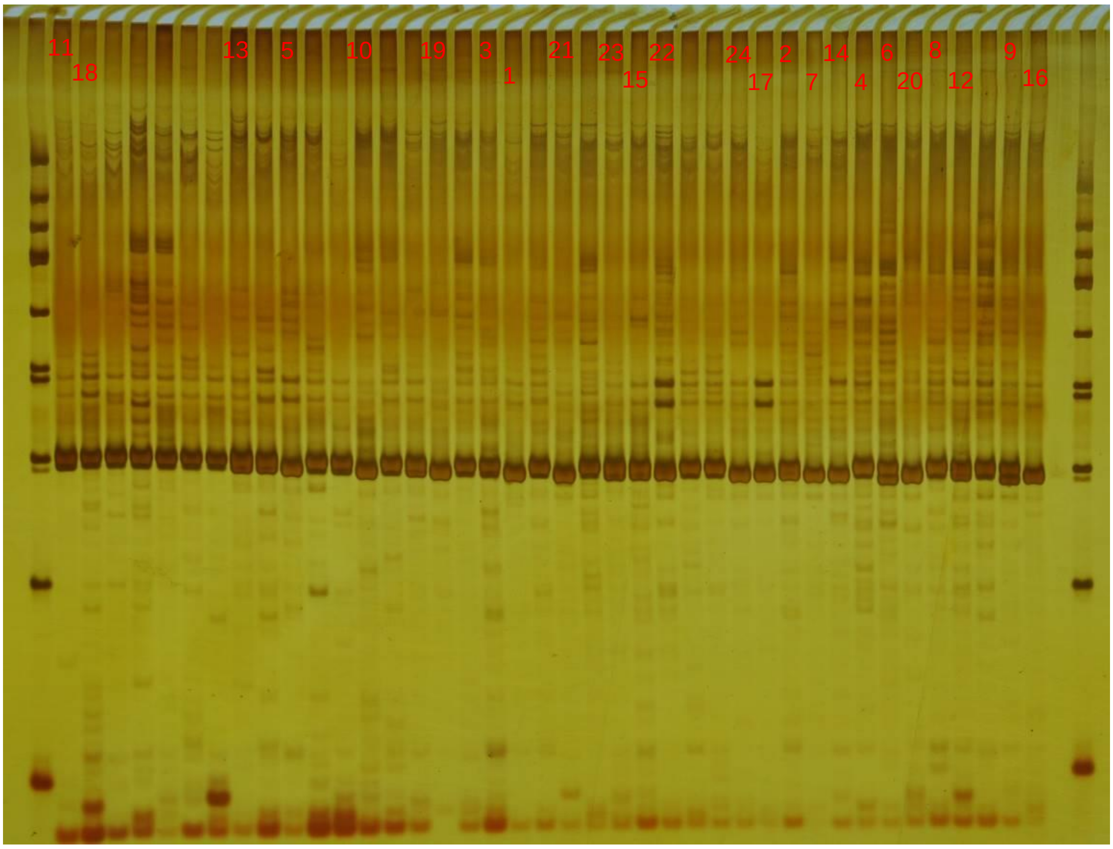

ESP-327

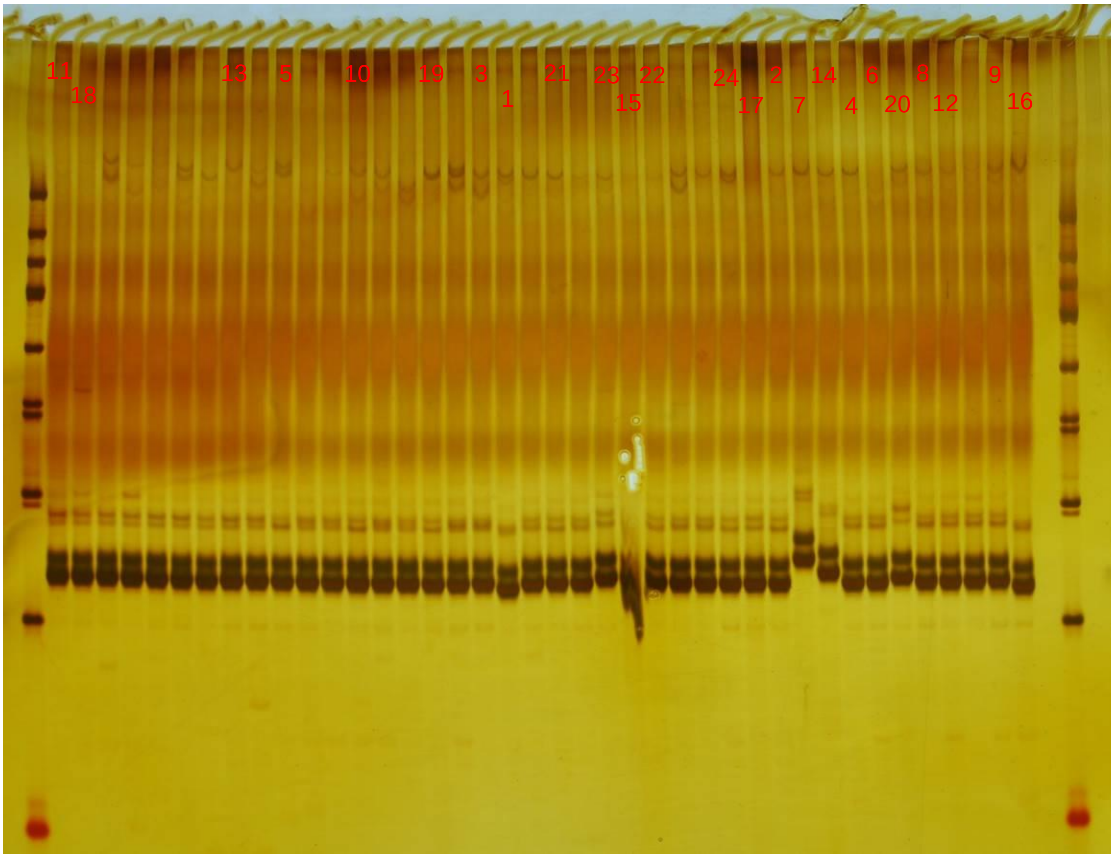

ESP-332

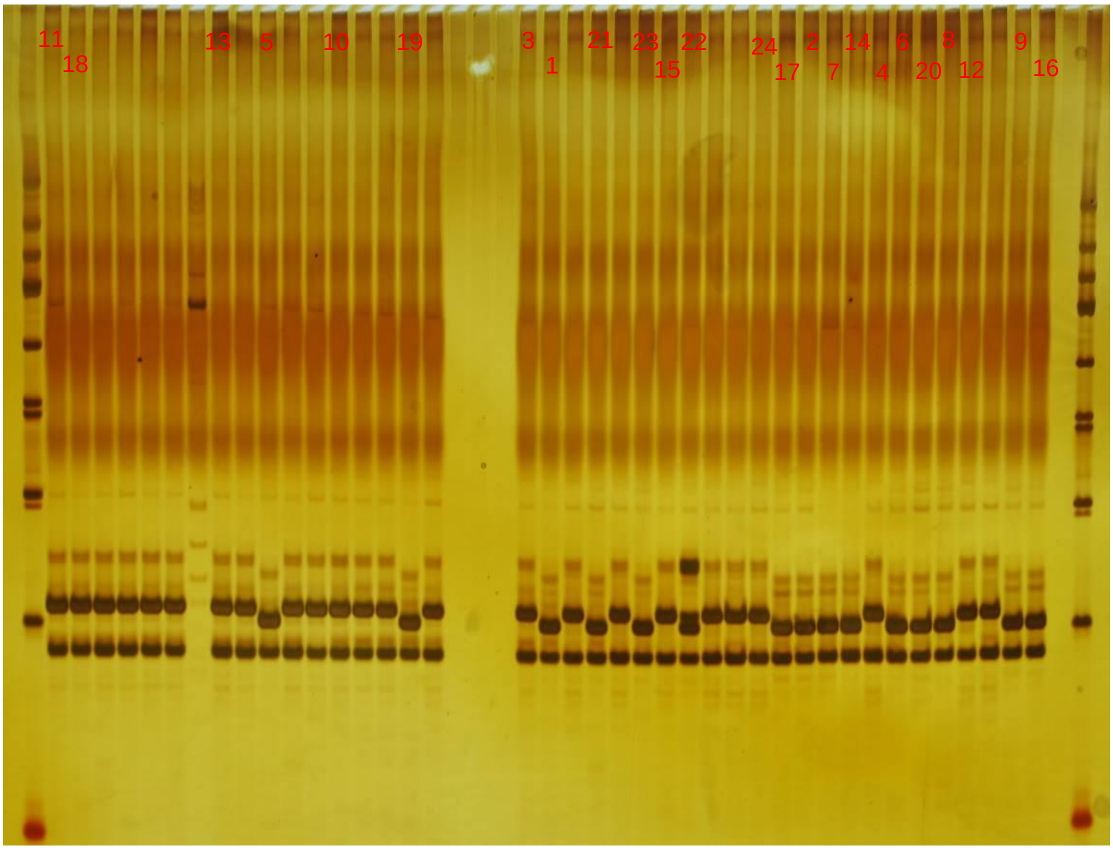

ESP-335

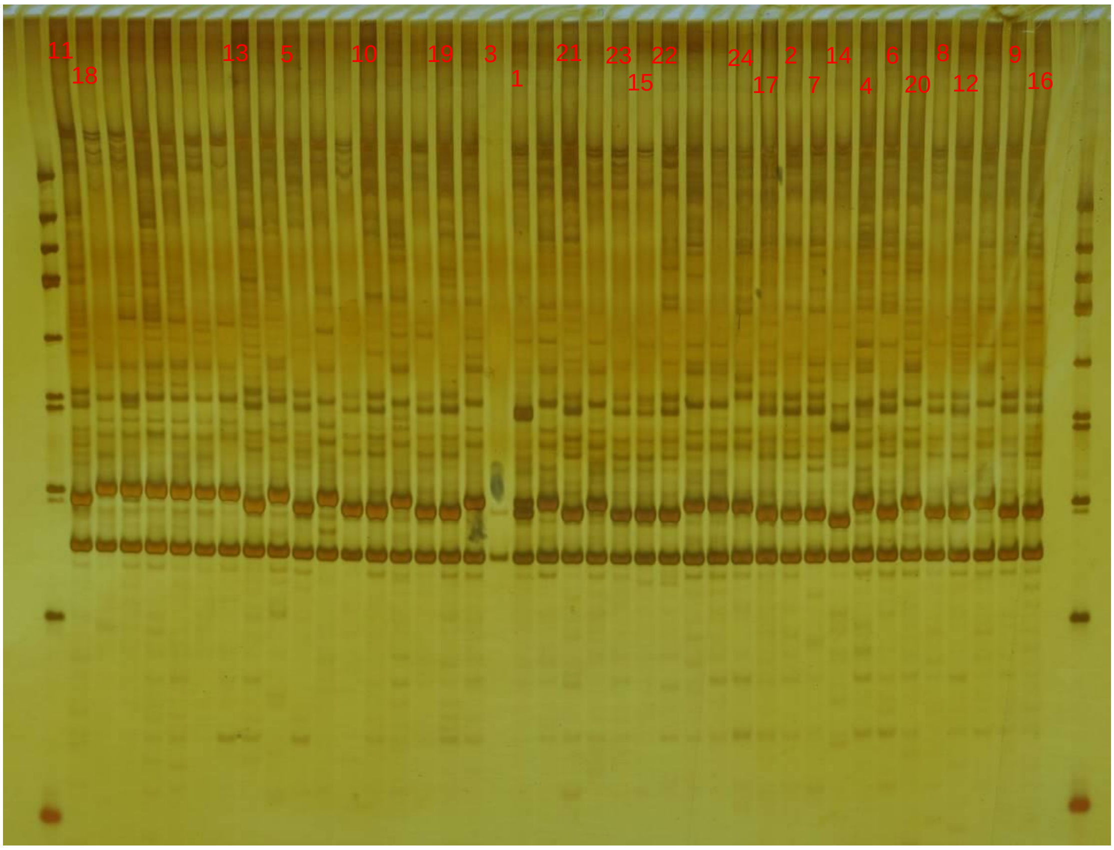

ESP-337

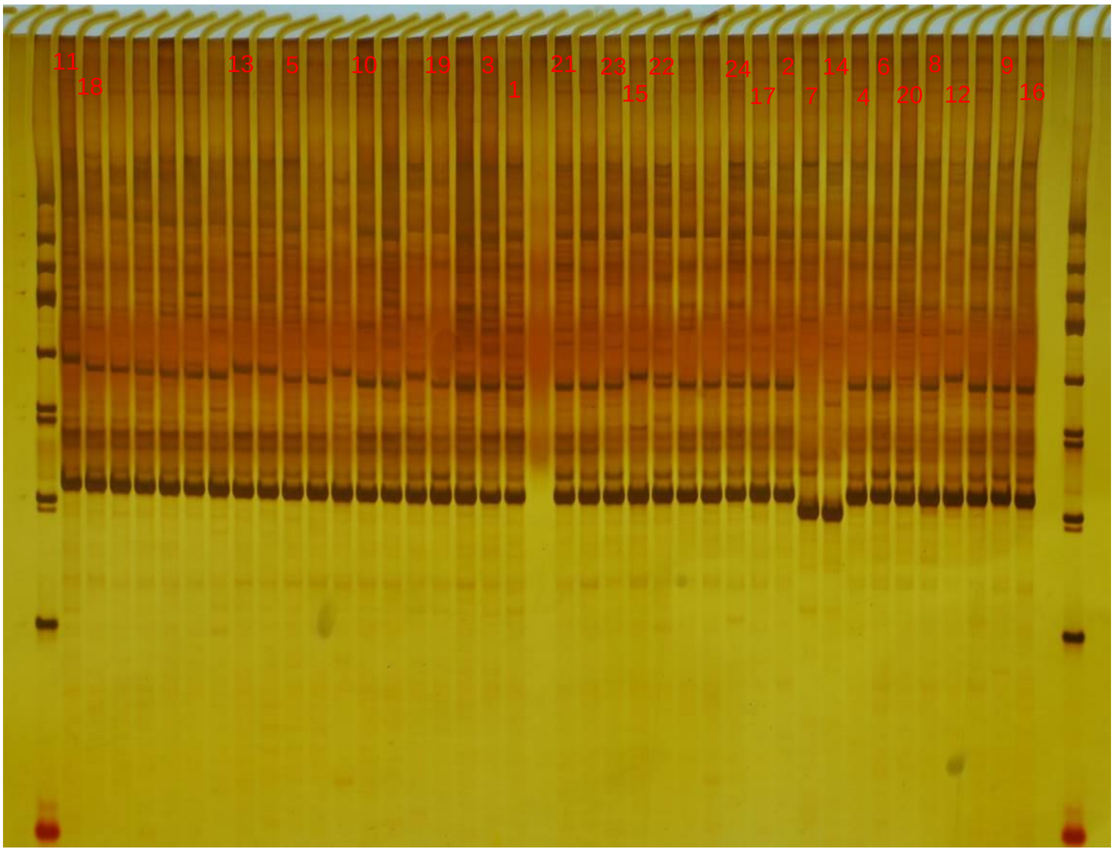

ESP-353

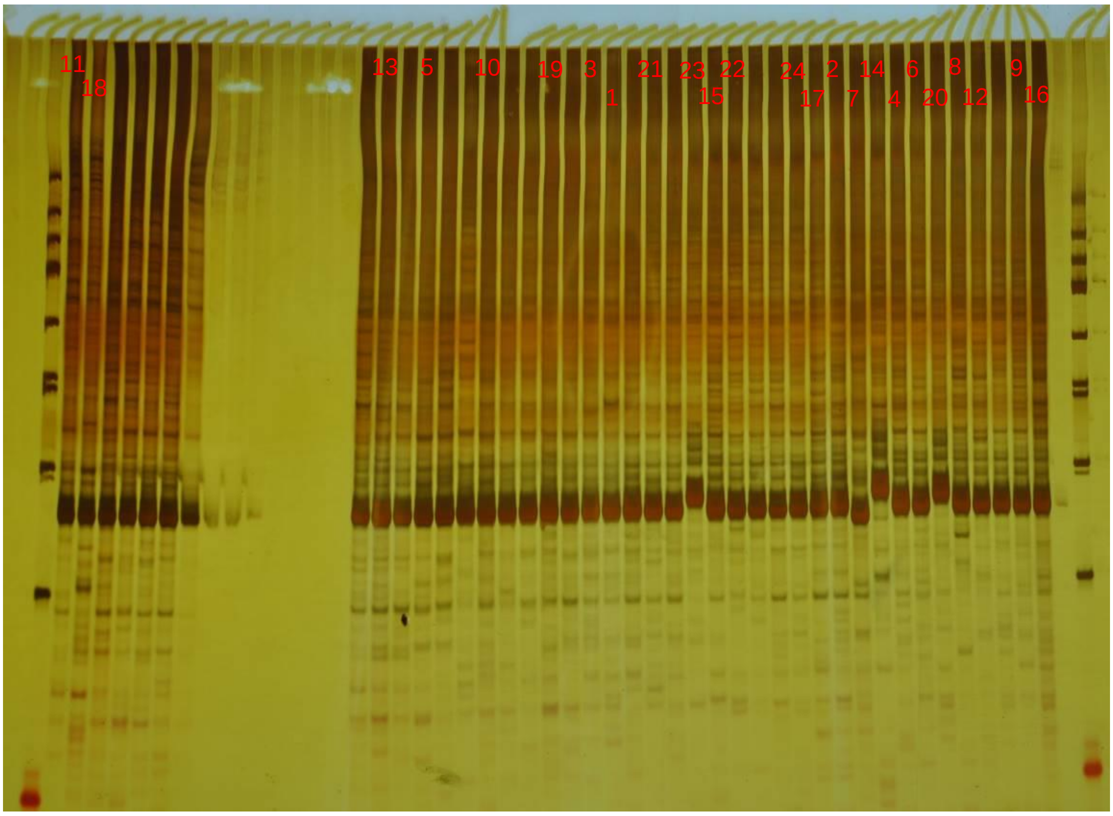

ESP-359

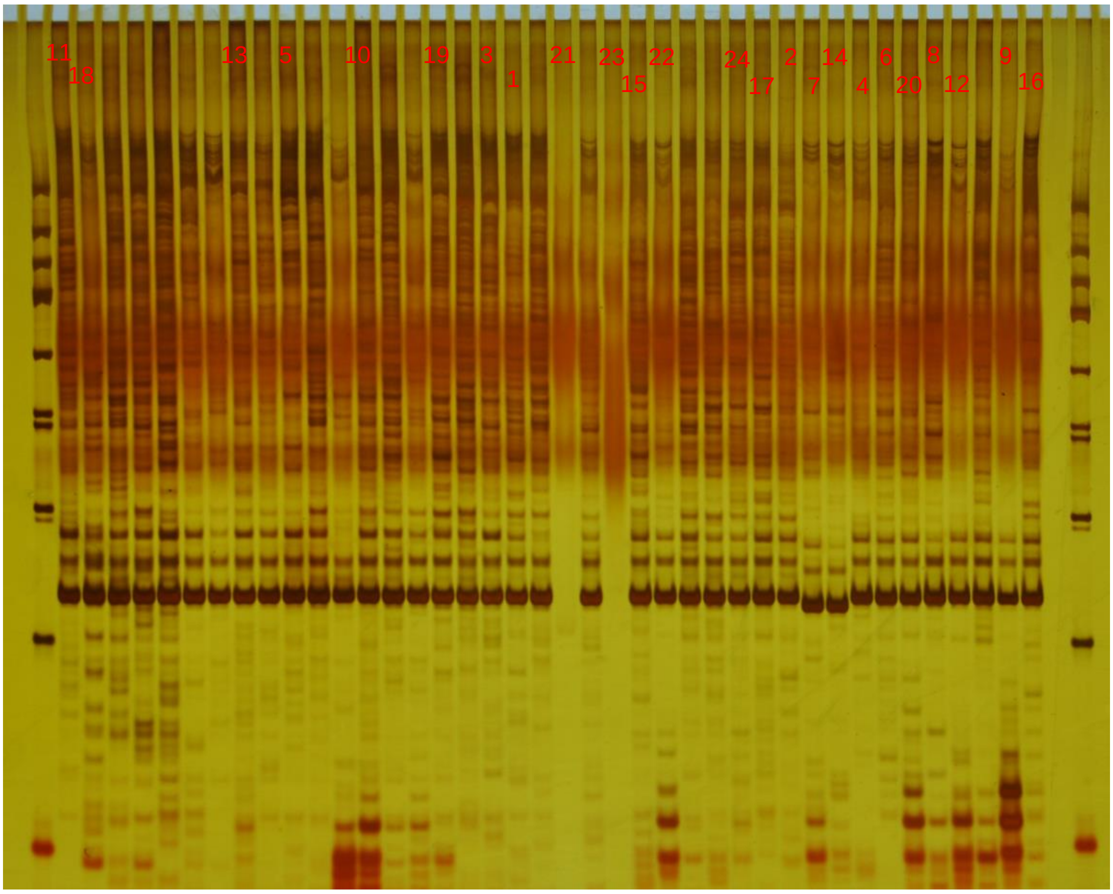

ESP-360

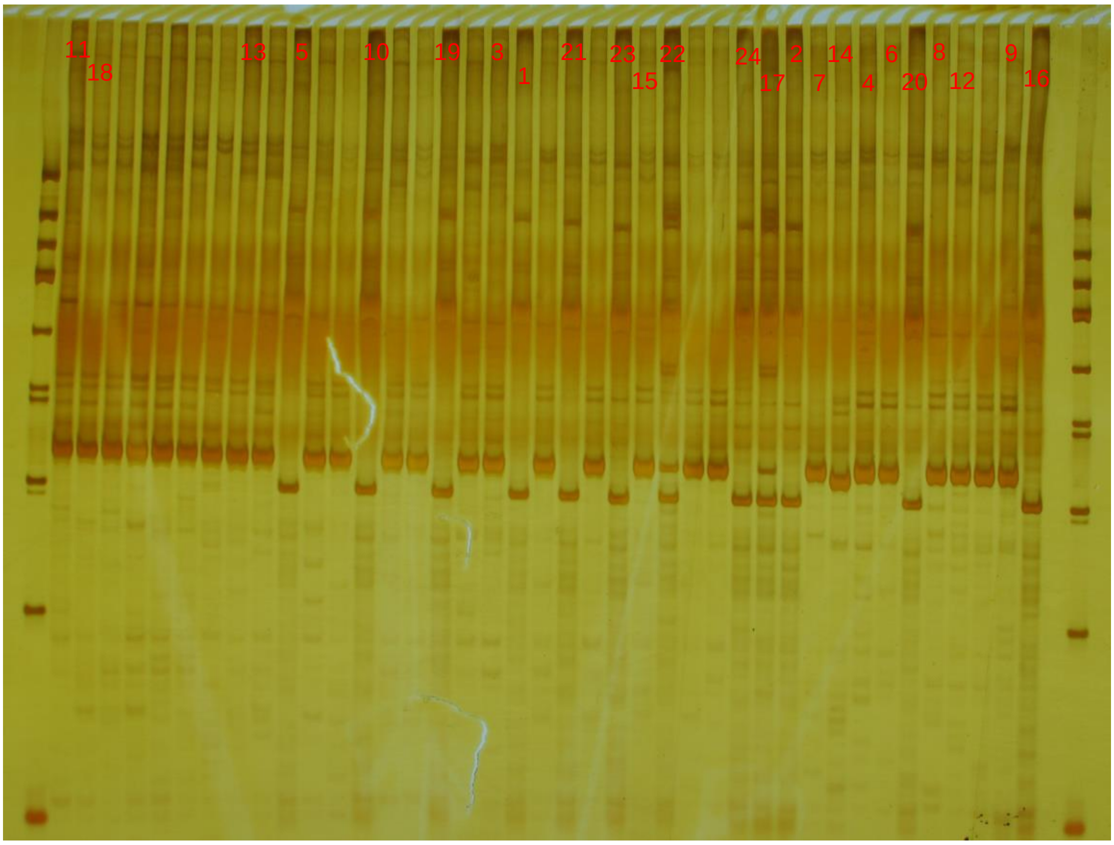

ESP-361

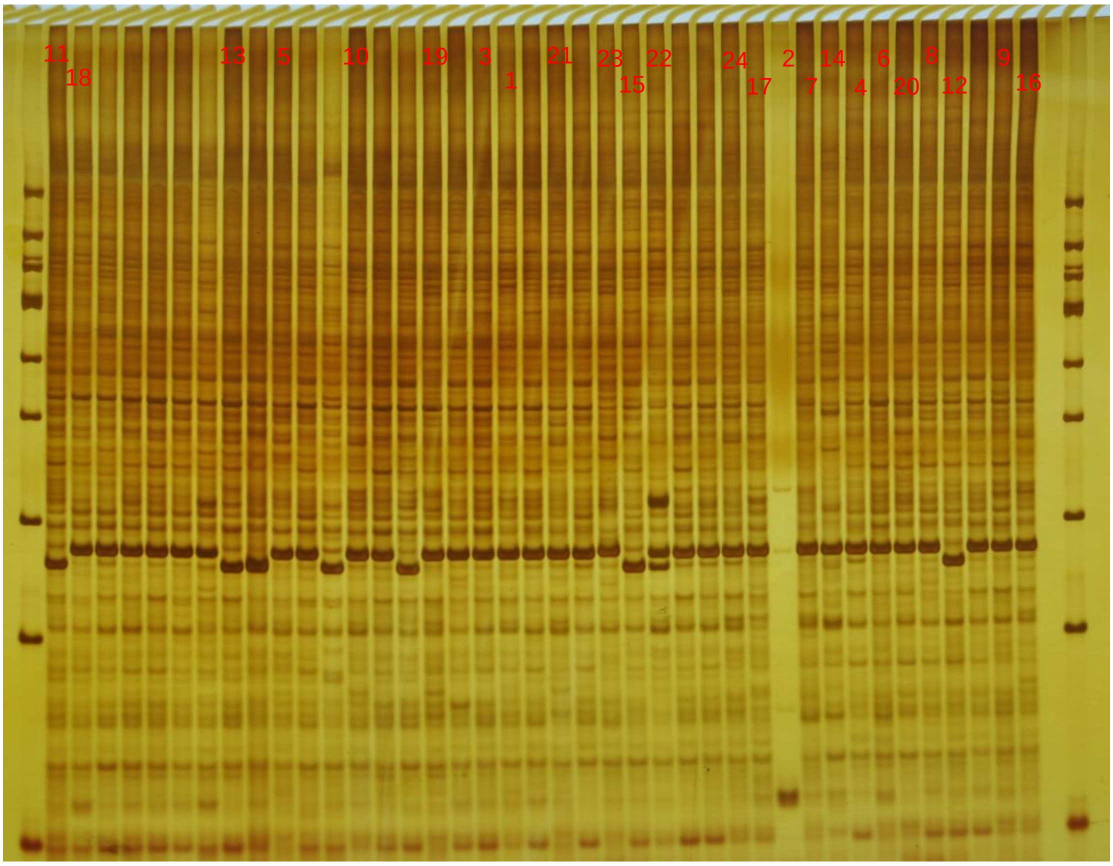

ESP-372

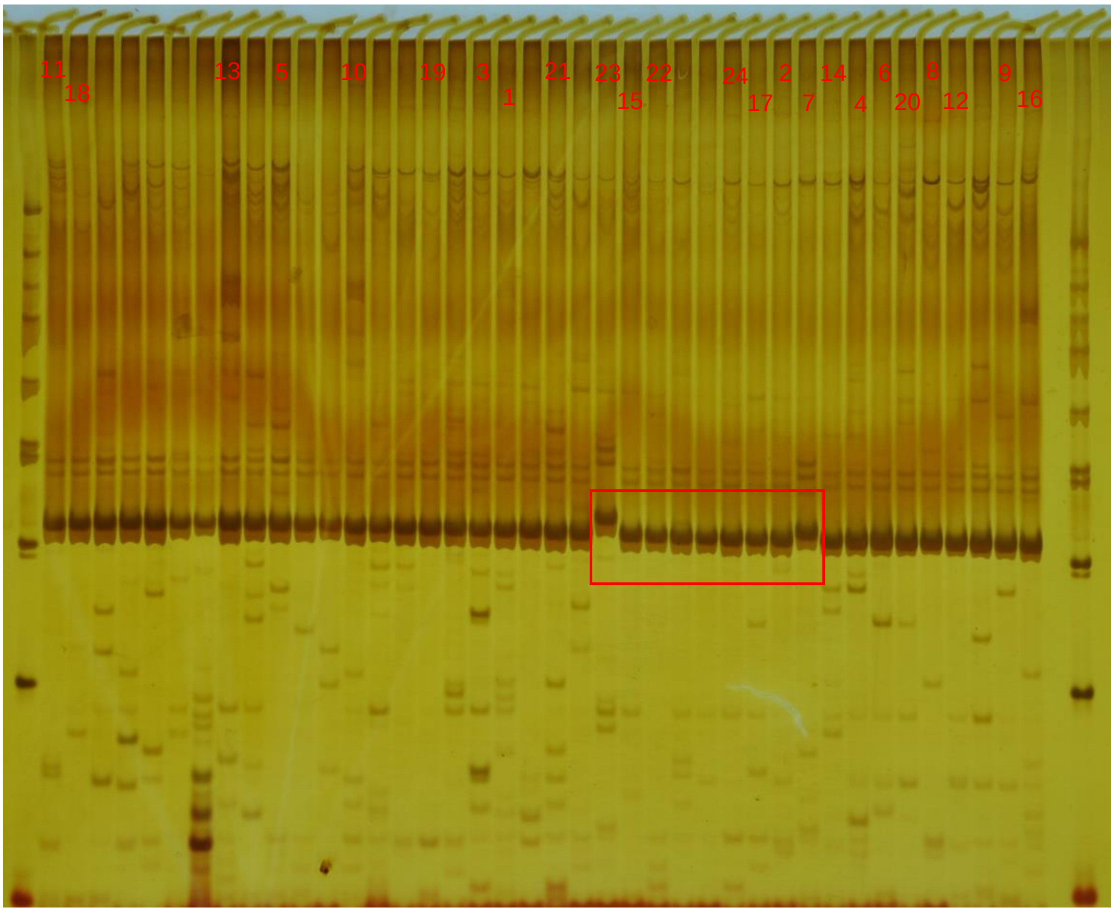

ESP-379

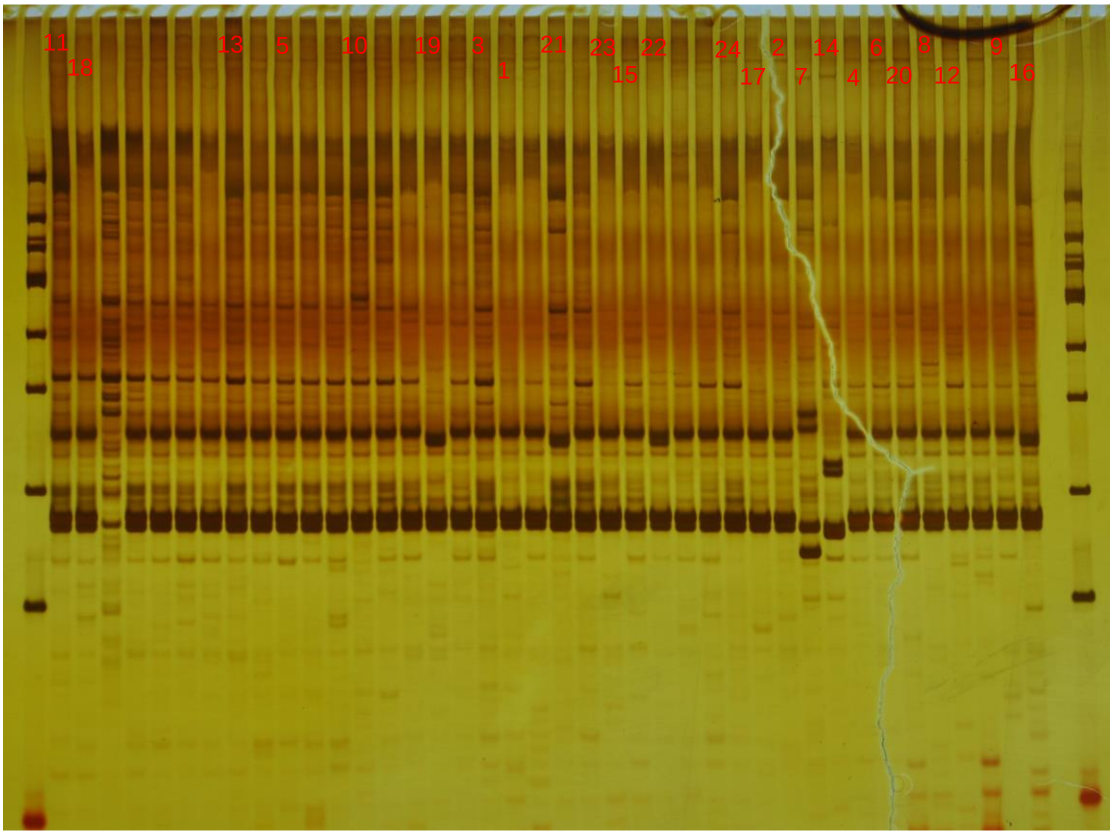

ESP-391

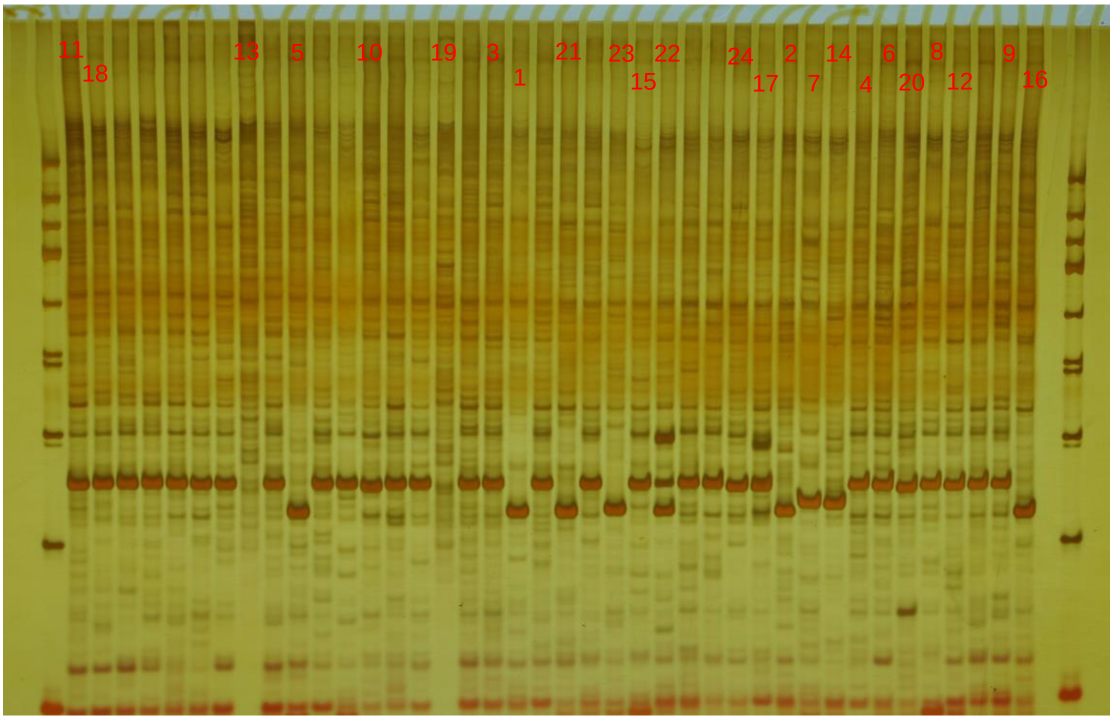

ESP-397

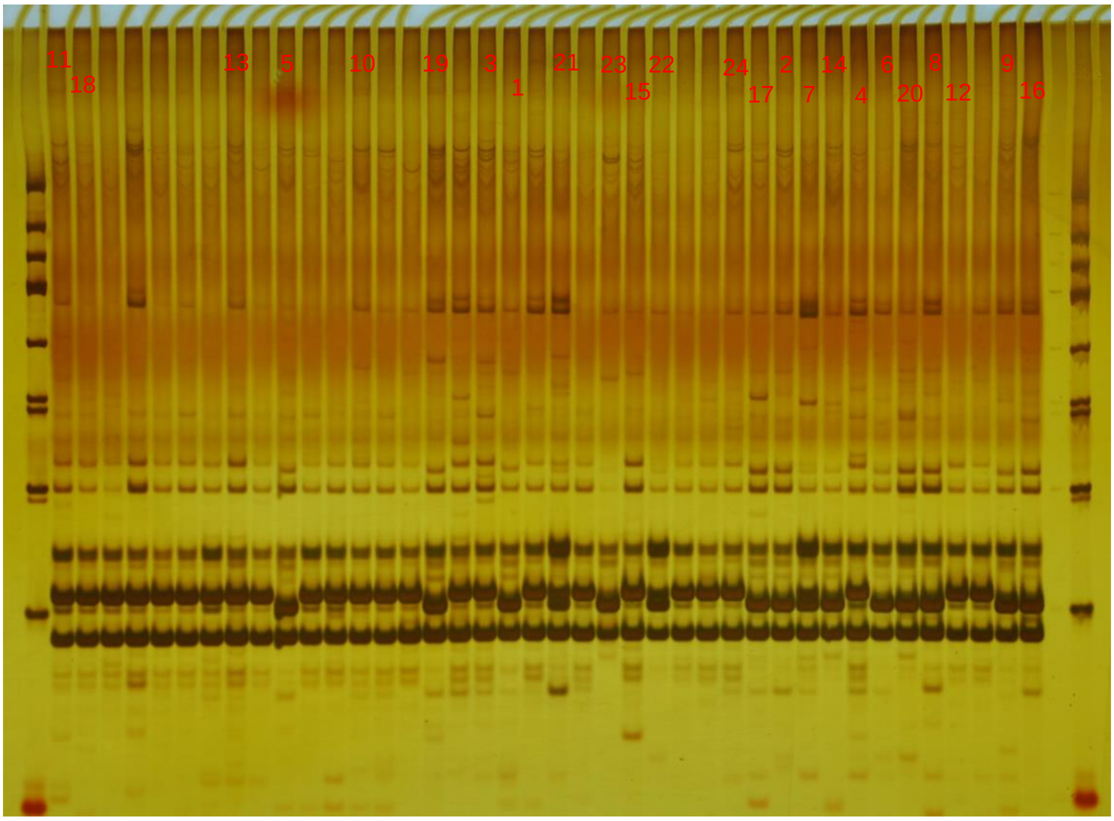

Fig. S5. The original and full-length gel images for amplification products of part polymorphic primers pairs on studied accessions. The accessions numbers showed in table 6 were labeled on each gel image, and the bands surrounded by red frames from ESP-178 and ESP-372 were verified by sequencing the amplified products. The markers were on both sides of each gel image.
