# Supplementary material for: Transcriptomic resources for prairie grass (Bromus catharticus): expressed transcripts, tissue-specific genes, and identification and validation of EST-SSR markers
Source: BMC Plant Biol. 2021 Jun 7;21:264. doi: 10.1186/s12870-021-03037-y (PMC8186225; doi:10.1186/s12870-021-03037-y)
Supplement: Supplementary file 6 — Additional file 6: Table S1. Repeat distribution of mono-, di-, tri-, quard-, penta- and hexa- nucleotides. [file 12870_2021_3037_MOESM6_ESM.docx]

**Table S1.** Repeat distribution of mono-, di-, tri-, quard-, penta- and hexa- nucleotides

| Number of repeats | Mono- | Di- | Tri- | Quard- | Penta- | Hexa- | Total | Percent |
| --- | --- | --- | --- | --- | --- | --- | --- | --- |
| 5 | 0 | 0 | 10,618 | 775 | 90 | 16 | 11,499 | 30.84% |
| 6 | 0 | 3,675 | 3,564 | 100 | 0 | 13 | 7,352 | 19.72% |
| 7 | 0 | 1,772 | 979 | 13 | 0 | 4 | 2,768 | 7.42% |
| 8 | 0 | 1,127 | 44 | 0 | 0 | 1 | 1,172 | 3.14% |
| 9 | 0 | 730 | 0 | 0 | 0 | 0 | 730 | 1.96% |
| 10 | 6,552 | 575 | 0 | 1 | 0 | 0 | 7,128 | 19.12% |
| 11 | 2,391 | 282 | 0 | 0 | 0 | 0 | 2,673 | 7.17% |
| 12 | 1,280 | 13 | 0 | 1 | 0 | 0 | 1,294 | 3.47% |
| 13 | 756 | 0 | 2 | 0 | 0 | 0 | 758 | 2.03% |
| 14 | 459 | 0 | 0 | 0 | 0 | 0 | 459 | 1.23% |
| 15 | 366 | 0 | 0 | 0 | 0 | 0 | 366 | 0.98% |
| 16 | 266 | 0 | 0 | 0 | 0 | 0 | 266 | 0.71% |
| 17 | 210 | 0 | 0 | 0 | 0 | 0 | 210 | 0.56% |
| 18 | 161 | 0 | 0 | 0 | 0 | 0 | 161 | 0.43% |
| 19 | 101 | 0 | 0 | 0 | 0 | 0 | 101 | 0.27% |
| 20 | 136 | 0 | 0 | 0 | 0 | 0 | 136 | 0.36% |
| 21 | 123 | 0 | 0 | 0 | 0 | 0 | 123 | 0.33% |
| 22 | 73 | 0 | 1 | 0 | 0 | 0 | 74 | 0.20% |
| 23 | 16 | 0 | 0 | 0 | 0 | 0 | 16 | 0.04% |
| 24 | 2 | 0 | 0 | 0 | 0 | 0 | 2 | 0.01% |
| Total | 12,892 | 8,174 | 15,208 | 890 | 90 | 34 | 37,288 | - |
| Percent | 34.57% | 21.92% | 40.79% | 2.39% | 0.24% | 0.09% | - | - |
