# Supplementary material for: Transcriptomic resources for prairie grass (Bromus catharticus): expressed transcripts, tissue-specific genes, and identification and validation of EST-SSR markers
Source: BMC Plant Biol. 2021 Jun 7;21:264. doi: 10.1186/s12870-021-03037-y (PMC8186225; doi:10.1186/s12870-021-03037-y)
Supplement: Supplementary file 7 — Additional file 7: Table S2. Details of 52 relevant EST-SSR markers. [file 12870_2021_3037_MOESM7_ESM.docx]

**Table S2.** Details of 52 relevant EST-SSR markers.

| No. | Primer code | Gene_id | SSR type | Forward primer (5'-3') | Tm (℃) | Reverse primer (3'-5') | Tm (℃) | Product size (bp) | NF | NPF | PP（%） | PIC | Rp | MI |
| --- | --- | --- | --- | --- | --- | --- | --- | --- | --- | --- | --- | --- | --- | --- |
| 1 | ESP-11 | CL-21662.170759 | (AG)8 | CGCGCCGTCAAATTAGACTG | 59.98 | CGCCTGTCTGAATGGAAGGT | 60.04 | 120 | 2 | 2 | 100.00 | 0.080 | 0.083 | 0.160 |
| 2 | ESP-27 | CL-21662.94142 | (TCC)5 | GCGGGACACCACAGGATTTA | 60.04 | TGGAGGAGGAGCAAAAGCTG | 59.96 | 141 | 2 | 2 | 100.00 | 0. 080 | 0.083 | 0.160 |
| 3 | ESP-72 | CL-21662.92738 | (CA)6 | TTCCATGCCAATCGCTGTCT | 60.04 | GACGCCAATTCAGAGCGAAC | 59.91 | 187 | 2 | 2 | 100.00 | 0.153 | 0.333 | 0.306 |
| 4 | ESP-127 | CL-21662.94935 | (CAG)6 | AACGGATCCGATTCCACCAC | 60.11 | GATCTCGTTGACTCCTGCCC | 60.18 | 182 | 4 | 2 | 50.00 | 0.196 | 1.000 | 0.196 |
| 5 | ESP-128 | CL-21662.185168 | (GTCC)5 | GTGACGGCGATGGGTATCAA | 60.18 | GTGTCGTCACCCGGTTAAGA | 59.69 | 255 | 4 | 4 | 100.00 | 0.148 | 0.667 | 0.590 |
| 6 | ESP-151 | CL-21662.100707 | (AGCA)5 | AGTTTGCTCCGGTTACAGGG | 59.96 | CATGCTTGCCGTGCTTGATT | 60.11 | 104 | 4 | 3 | 75.00 | 0.263 | 1.750 | 0.592 |
| 7 | ESP-164 | CL-21662.98500 | (TGCT)5 | TTGCGGAGGTGGAGATGAAC | 60.04 | AGGCAAGTTCGTGCTAGTGA | 59.32 | 267 | 2 | 2 | 100.00 | 0.500 | 2.000 | 1.000 |
| 8 | ESP-166 | CL-21662.76086 | (CAGT)5 | GGTGTCGGAAGCCTTTGAGA | 59.97 | CGTTTCCCTAGGCAGACTCC | 59.82 | 269 | 3 | 2 | 66.67 | 0.263 | 1.083 | 0.350 |
| 9 | ESP-178 | CL-21662.66171 | (CGC)6 | CCAAACCAAACCAAACCGCA | 60.11 | GAAGAGAGCGGGGTCTATGC | 59.97 | 135 | 7 | 7 | 100.00 | 0.210 | 2.000 | 1.472 |
| 10 | ESP-179 | CL-21662.42236 | (AGTC)5 | GGTGTCGGAAGCCTTTGAGA | 59.97 | CCGTGTCATCGTTCCCTAGG | 59.90 | 260 | 2 | 2 | 100.00 | 0.413 | 1.167 | 0.826 |
| 11 | ESP-182 | CL-21662.109155 | (GCAC)5 | CCGGGATCTCGTTAGCTCAC | 59.97 | CACAGGCCTCCAGTCAGTC | 59.71 | 132 | 2 | 2 | 100.00 | 0.486 | 1.667 | 0.972 |
| 12 | ESP-190 | CL-21662.69773 | (GTGA)5 | CGAGATCGACAACGCAGAGA | 59.90 | CCAGTACATGGCATGACGTCT | 60.14 | 206 | 5 | 5 | 100.00 | 0.261 | 2.167 | 1.306 |
| 13 | ESP-193 | CL-21662.78504 | (GTGA)5 | GGACCTGTGACCTGTGAGTG | 59.97 | GTCAGTGAGTGATCGGTTGA | 57.28 | 157 | 3 | 2 | 66.67 | 0.220 | 0.833 | 0.293 |
| 14 | ESP-202 | CL-21662.72253 | (GGC)6 | ATGAGGGAGCGGTGTTCGTA | 60.97 | TGTCATCCATCCAACCACGG | 60.04 | 230 | 3 | 3 | 100.00 | 0.358 | 1.917 | 1.073 |
| 15 | ESP-215 | CL-21662.143948 | (GCAC)5 | CCGGGATCTCGTTAGCTCAC | 59.97 | CACAGGCCTCCAGTCAGTC | 59.71 | 132 | 2 | 2 | 100.00 | 0.486 | 1.667 | 0.972 |
| 16 | ESP-242 | CL-21662.132804 | (CAG)6 | GGCGGACGACAAATGCATC | 59.94 | TCCTCGACTTGGACTTGCAC | 59.97 | 275 | 2 | 1 | 50.00 | 0.250 | 1.000 | 0.125 |
| 17 | ESP-248 | CL-21662.158057 | (AC)8 | GCTGCTCAACTCCTCTACGG | 60.18 | ACACAGTCATACACGCCACA | 59.61 | 174 | 4 | 3 | 75.00 | 0.172 | 0.833 | 0.387 |
| 18 | ESP-251 | CL-21662.50749 | (TTCG)5 | CAGCCGCCCAGATAGCCC | 62.63 | GAGTGGGACTGAAGAGTGGC | 60.04 | 134 | 2 | 2 | 100.00 | 0.304 | 0.750 | 0.608 |
| 19 | ESP-252 | CL-21662.70253 | (GAGG)5 | CCGGCCGAGACCAGAAATAA | 59.82 | TCTCGATCTCCTCTCGTCCC | 59.89 | 136 | 3 | 3 | 100.00 | 0.354 | 1.833 | 1.063 |
| 20 | ESP-289 | CL-21662.65918 | (GCG)6 | GTCTACAGAGGTCTCGGGGT | 60.03 | GAAGCCTGTCTTTCTCCCCC | 60.04 | 116 | 4 | 4 | 100.00 | 0.177 | 0.833 | 0.708 |
| 21 | ESP-302 | CL-21662.4957 | (TTCG)5 | CAGCCGCCCAGATAGCCC | 62.63 | GAGTGGGACTGAAGAGTGGC | 60.04 | 134 | 2 | 2 | 100.00 | 0.278 | 0.667 | 0.556 |
| 22 | ESP-304 | CL-21662.7567 | (ATAC)5 | TTCCGCCAATATTTCGCCCT | 60.11 | ACTAGTGCAAGCAGAGCCTC | 59.75 | 210 | 5 | 4 | 80.00 | 0.270 | 1.917 | 0.864 |
| 23 | ESP-305 | CL-21662.50290 | (GAAG)5 | CACCACTGGATGCATGCATG | 59.90 | ACGTACGAGGTCAATGGCAG | 60.11 | 132 | 2 | 2 | 100.00 | 0.080 | 0.167 | 0.160 |
| 24 | ESP-306 | CL-21662.96245 | (CCGA)5 | GAAGCCCAAACCCTAACCCT | 59.59 | GCGTACTCGACCTGGAAGAG | 59.90 | 119 | 6 | 5 | 83.33 | 0.102 | 0.667 | 0.424 |
| 25 | ESP-311 | CL-21662.118533 | (AAG)6 | GATCCAGGTTAGCCAGCTCC | 59.89 | GGAACGATCACGAGCAAGTA | 57.73 | 122 | 4 | 3 | 75.00 | 0.109 | 0.500 | 0.246 |
| 26 | ESP-313 | CL-21662.23153 | (GAG)6 | AACAACAACAACGATGGCGG | 59.97 | CGGTGAGAGTGTGTGGTTGA | 59.90 | 228 | 3 | 2 | 66.67 | 0.100 | 0.333 | 0.133 |
| 27 | ESP-319 | CL-46897.0 | (AGTG)5 | CTTTCCTGAGCTGCTCCACA | 59.96 | ACCCTATCTCCAGCCCTGAT | 59.43 | 150 | 2 | 2 | 100.00 | 0.497 | 1.833 | 0.993 |
| 28 | ESP-322 | CL-21662.74021 | (CAT)7 | TTCGCCTCTATCATCGCCAC | 59.97 | CGTCCGGGGACTTTCAAAGA | 59.97 | 222 | 7 | 7 | 100.00 | 0.281 | 3.000 | 1.965 |
| 29 | ESP-327 | CL-21662.93540 | (TG)10 | ACCTTGGAAATGTGCAACGC | 59.97 | GCGTCGTAGTCAGCTATGCT | 59.97 | 114 | 5 | 5 | 100.00 | 0.168 | 1.000 | 0.840 |
| 30 | ESP-332 | CL-21662.100705 | (AGCA)5 | AGTTTGCTCCGGTTACAGGG | 59.96 | CATGCTTGCCGTGCTTGATT | 60.11 | 104 | 3 | 2 | 66.67 | 0.318 | 1.583 | 0.424 |
| 31 | ESP-335 | CL-21662.140391 | (GCGA)5 | CGGTTTGATTCGAAACGCCC | 60.45 | GAACCCTAGCTCGCAGTCG | 60.23 | 191 | 4 | 3 | 75.00 | 0.208 | 1.083 | 0.467 |
| 32 | ESP-337 | CL-21662.21181 | (CATC)5 | AGACATCCTCTTCCCCCGAA | 59.96 | ATGCGAAGGAGTGATGGACC | 59.82 | 155 | 4 | 4 | 100.00 | 0.249 | 1.250 | 0.997 |
| 33 | ESP-338 | CL-21662.11926 | (ACG)6 | ATGGACTTCATGCTCCGAGG | 59.53 | GGGATCGAAGAAGGCGTTCC | 60.81 | 213 | 3 | 2 | 66.67 | 0.053 | 0.167 | 0.071 |
| 34 | ESP-341 | CL-21662.109095 | (AGTT)6 | AGTCTTTAATTTGGTCGTGCGC | 60.10 | AAGCAAGAGACACCAGCTGG | 60.25 | 215 | 4 | 4 | 100.00 | 0.195 | 1.083 | 0.781 |
| 35 | ESP-351 | CL-21662.132002 | (CAT)7 | TTCGCCTCTATCATCGCCAC | 59.97 | CGTCCGGGGACTTTCAAAGA | 59.97 | 222 | 4 | 4 | 100.00 | 0.276 | 1.833 | 1.104 |
| 36 | ESP-352 | CL-21662.148875 | (ATCG)5 | CCATCCAATCCACCTCGACG | 60.53 | CGCATACCATCCCCGAGAAA | 59.89 | 203 | 2 | 2 | 100.00 | 0.497 | 1.833 | 0.993 |
| 37 | ESP-353 | CL-21662.70164 | (TC)9 | ATGAACTGGAAGAGCTCGGC | 60.11 | CCCACAGGCAACCAACAAAG | 59.90 | 129 | 3 | 3 | 100.00 | 0.192 | 0.667 | 0.576 |
| 38 | ESP-359 | CL-21662.153304 | (AGC)6 | AAGAGGAAGTTCGCCGTAGC | 60.11 | CGCCCTTGTACGTCCTCATC | 60.53 | 115 | 2 | 2 | 100.00 | 0.153 | 0.333 | 0.306 |
| 39 | ESP-360 | CL-21662.65494 | (ACAG)6 | TGGTTATCTGACAAGCGGTGT | 59.65 | GCATGGTGCATGATGATGGG | 59.68 | 165 | 3 | 3 | 100.00 | 0.360 | 2.083 | 1.080 |
| 40 | ESP-361 | CL-21662.89037 | (TTCG)5 | CAGCCGCCCAGATAGCCC | 62.63 | GAGTGGGACTGAAGAGTGGC | 60.04 | 134 | 2 | 2 | 100.00 | 0.278 | 0.667 | 0.556 |
| 41 | ESP-363 | CL-21662.88167 | (AGTT)6 | AGTCTTTAATTTGGTCGTGCGC | 60.10 | AAGCAAGAGACACCAGCTGG | 60.25 | 215 | 4 | 4 | 100.00 | 0.230 | 1.250 | 0.920 |
| 42 | ESP-369 | CL-21662.105115 | (ACG)7 | ATCTGTCTTCAGCACCACCG | 60.04 | GAAGATGGGGCTCAAGAGGG | 59.82 | 226 | 2 | 2 | 100.00 | 0.153 | 0.333 | 0.306 |
| 43 | ESP-370 | CL-21662.140791 | (AGA)6 | CCTGCTCGAAATCACTCGGA | 59.83 | TCTCTCCTCCGACGACGAAT | 60.11 | 219 | 2 | 2 | 100.00 | 0.444 | 1.333 | 0.889 |
| 44 | ESP-372 | CL-45072.1 | (TCCA)5 | GCCAAACCAGCTTAAGACGC | 60.11 | AGGGGTTTGCACGTATTGGT | 59.89 | 156 | 3 | 3 | 100.00 | 0.104 | 0.333 | 0.313 |
| 45 | ESP-379 | CL-21662.110154 | (GGAT)5 | CGTGAACACCAGCTTCCTCT | 59.97 | AATCTCTCCATCCATCGGCC | 59.31 | 134 | 3 | 3 | 100.00 | 0.104 | 0.333 | 0.313 |
| 46 | ESP-385 | CL-21662.71248 | (CTT)7 | GTGGAGAATCCGAGCACCAA | 60.04 | CTGGGAACTTAGCTAGCCGG | 59.90 | 149 | 2 | 2 | 100.00 | 0.219 | 0.500 | 0.438 |
| 47 | ESP-391 | CL-21662.28348 | (CACG)5 | GAAGCCATGAAAAGTCGCCG | 60.18 | ATGGACAGTGATGTGGGCTG | 60.04 | 125 | 3 | 3 | 100.00 | 0.351 | 1.583 | 1.052 |
| 48 | ESP-397 | CL-21662.102025 | (AGCA)5 | AGTTTGCTCCGGTTACAGGG | 59.96 | CATGCTTGCCGTGCTTGATT | 60.11 | 104 | 3 | 2 | 66.67 | 0.313 | 1.500 | 0.417 |
| 49 | ESP-406 | CL-21662.93725 | (CAT)7 | TTCGCCTCTATCATCGCCAC | 59.97 | CGTCCGGGGACTTTCAAAGA | 59.97 | 222 | 3 | 3 | 100.00 | 0.348 | 1.750 | 1.045 |
| 50 | ESP-415 | CL-21662.24293 | (GCC)6 | GAAACGGTACCTCTGAGCCC | 60.11 | GAAGTAGGCGCGGAAGAGAA | 59.83 | 174 | 2 | 2 | 100.00 | 0.491 | 1.750 | 0.983 |
| 51 | ESP-417 | CL-21662.82463 | (ACG)7 | ATCTGTCTTCAGCACCACCG | 60.04 | GAAGATGGGGCTCAAGAGGG | 59.82 | 226 | 2 | 2 | 100.00 | 0.153 | 0.333 | 0.306 |
| 52 | ESP-419 | CL-21662.185820 | (ACTC)5 | CTGCTCTGAAGAAACCGGGT | 59.97 | TGAGCCACGGGAGAGAGTAA | 59.96 | 192 | 5 | 5 | 100.00 | 0.267 | 2.000 | 1.333 |

NF- Number of fragments; NPF- Number of polymorphic fragments; PP- Percent of polymorphic fragments; PIC- Polymorphic information content; Rp- Resolving power; MI- Marker index
