# Supplementary material for: Transcriptomic resources for prairie grass (Bromus catharticus): expressed transcripts, tissue-specific genes, and identification and validation of EST-SSR markers
Source: BMC Plant Biol. 2021 Jun 7;21:264. doi: 10.1186/s12870-021-03037-y (PMC8186225; doi:10.1186/s12870-021-03037-y)
Supplement: Supplementary file 8 — Additional file 8: Table S3. Descriptive statistics of 11 traits for 24 B. catharticus accessions. [file 12870_2021_3037_MOESM8_ESM.docx]

**Table S3.** Descriptive statistics of 11 traits for 24 *B. catharticus* accessions.

| Accession codes | FMY | DMY | PH | FLL | PLL | LFI | FLW | PLW | SD | TN | DSH |
| --- | --- | --- | --- | --- | --- | --- | --- | --- | --- | --- | --- |
| PI309957 | 1.42 | 0.35 | 158.12 | 37.69 | 44.04 | 77.13 | 12.35 | 14.00 | 6.09 | 78.22 | 199 |
| PI197848 | 1.74 | 0.43 | 167.07 | 36.87 | 42.92 | 64.21 | 13.61 | 15.60 | 8.35 | 49.67 | 202 |
| PI331171 | 1.25 | 0.31 | 156.95 | 35.96 | 43.28 | 77.19 | 12.36 | 14.10 | 6.39 | 71.44 | 200 |
| PI331172 | 1.38 | 0.34 | 145.06 | 34.14 | 39.54 | 62.85 | 11.87 | 13.63 | 7.00 | 67.89 | 200 |
| PI308506 | 0.83 | 0.20 | 115.20 | 24.97 | 33.04 | 90.21 | 7.80 | 8.91 | 3.70 | 125.56 | 217 |
| PI309955 | 1.35 | 0.33 | 161.55 | 34.91 | 42.55 | 65.34 | 12.83 | 14.50 | 7.63 | 57.33 | 203 |
| PI309958 | 1.42 | 0.35 | 152.76 | 34.84 | 41.23 | 63.46 | 13.37 | 15.86 | 8.05 | 50.89 | 203 |
| PI204157 | 1.26 | 0.31 | 153.47 | 33.67 | 41.30 | 60.19 | 11.91 | 14.12 | 7.60 | 52.89 | 203 |
| PI292258 | 1.54 | 0.38 | 162.56 | 36.34 | 42.66 | 58.23 | 13.20 | 14.75 | 8.44 | 54.78 | 203 |
| PI413792 | 1.69 | 0.41 | 154.29 | 36.47 | 44.80 | 68.12 | 13.74 | 16.42 | 7.25 | 75.22 | 203 |
| PI634281 | 1.37 | 0.33 | 158.64 | 33.50 | 38.76 | 69.54 | 11.57 | 13.89 | 6.55 | 71.67 | 199 |
| PI618750 | 1.53 | 0.37 | 144.82 | 32.56 | 40.75 | 76.15 | 11.93 | 14.04 | 6.17 | 88.78 | 203 |
| PI187000 | 1.35 | 0.33 | 153.20 | 36.35 | 40.74 | 70.54 | 12.66 | 15.61 | 6.86 | 65.44 | 199 |
| PI595114 | 1.41 | 0.34 | 161.09 | 36.72 | 42.42 | 62.02 | 12.69 | 14.75 | 7.00 | 73.44 | 196 |
| PI595118 | 1.46 | 0.36 | 128.07 | 34.91 | 39.91 | 70.78 | 13.43 | 15.61 | 5.24 | 105.44 | 197 |
| PI595115 | 1.33 | 0.33 | 155.57 | 34.24 | 39.34 | 65.85 | 12.41 | 15.40 | 7.55 | 60.67 | 196 |
| W610415 | 1.55 | 0.38 | 147.26 | 41.83 | 48.04 | 67.48 | 12.31 | 14.29 | 6.26 | 83.89 | 199 |
| PI409139 | 1.41 | 0.34 | 148.34 | 40.26 | 44.36 | 79.52 | 10.59 | 12.20 | 5.00 | 84.22 | 192 |
| PI377533 | 1.62 | 0.40 | 159.53 | 35.39 | 43.52 | 69.50 | 13.19 | 15.44 | 6.88 | 70.00 | 202 |
| Jiangxia | 1.48 | 0.36 | 153.41 | 37.52 | 43.42 | 70.63 | 12.21 | 14.66 | 6.35 | 80.56 | 201 |
| PI442077 | 0.99 | 0.24 | 126.18 | 37.34 | 42.48 | 60.69 | 14.28 | 13.70 | 5.21 | 54.56 | 206 |
| PI217593 | 1.66 | 0.41 | 161.05 | 35.54 | 42.67 | 77.19 | 12.92 | 15.31 | 7.56 | 71.00 | 201 |
| PI495807 | 1.41 | 0.34 | 164.37 | 36.04 | 44.03 | 62.72 | 12.72 | 14.65 | 7.61 | 48.78 | 202 |
| BCS1103 | 1.76 | 0.43 | 173.83 | 38.64 | 46.50 | 70.84 | 13.19 | 15.62 | 7.74 | 68.22 | 202 |
| Mean | 1.42 | 0.35 | 152.60 | 35.70 | 42.18 | 69.18 | 12.46 | 14.46 | 6.77 | 71.27 | 201 |
| Minimum | 0.83 | 0.20 | 115.20 | 24.97 | 33.04 | 58.23 | 7.80 | 8.91 | 3.70 | 48.78 | 192. |
| Maximum | 1.76 | 0.43 | 173.83 | 41.83 | 48.04 | 90.21 | 14.28 | 16.42 | 8.44 | 125.56 | 217 |
| Standard deviation | 0.21 | 0.05 | 13.12 | 3.04 | 2.88 | 7.34 | 1.25 | 1.47 | 1.13 | 17.73 | 4.44 |
| Coefficient of variation | 0.1466 | 0.1466 | 0.0860 | 0.0851 | 0.0682 | 0.1061 | 0.0999 | 0.1019 | 0.1663 | 0.2487 | 0.0221 |

PH- Plant height; FL- First leaf length; PL- Penultimate leaf length; LFI- Length of first internode; FLW- Flag leaf width; PLW- Penultimate leaf width; SD- Stem diameter; TN- Tiller number; FMY- Fresh matter yield; DMY- Dry matter yield; DSH- Days from seeding to heading.
