# Supplementary material for: Transcriptomic resources for prairie grass (Bromus catharticus): expressed transcripts, tissue-specific genes, and identification and validation of EST-SSR markers
Source: BMC Plant Biol. 2021 Jun 7;21:264. doi: 10.1186/s12870-021-03037-y (PMC8186225; doi:10.1186/s12870-021-03037-y)
Supplement: Supplementary file 9 — Additional file 9: Table S4. Details of eleven genes and their primers for qRT-PCR analysis. [file 12870_2021_3037_MOESM9_ESM.docx]

**Table S4.** Details of eleven genes and their primers for qRT-PCR analysis.

| No. | Gene ID | Primer sequence（5'-3'） | Annotation |
| --- | --- | --- | --- |
| 1 | Cluster-21662.39788 | F: CCGACGAGAGGAAGGTGGTG | Predicted: carotenoid cleavage dioxygenase 8 homolog A |
|  |  | R: GGACTTGATGAGGCTGTTGATGC |  |
| 2 | Cluster-21662.151963 | F: CTTCGGAAATAATCGCATCGTAGG | Receptor-like protein 12 |
|  |  | R: GGTTGGATCTCAAGACAAGGACAC |  |
| 3 | Cluster-21662.108468 | F: GTCGGCAAGAAGGGCAACG | ATP synthase subunit gamma |
|  |  | R: TGAGTAGATGAGGTCGCAGATGG |  |
| 4 | Cluster-21662.88693 | F: CCAGAGCAGCAGCATCAAGAAC | PsbP domain-containing protein 4 |
|  |  | R: TTGTGACGAACAGAGAAGAAGAGC |  |
| 5 | Cluster-21662.136127 | F: TGCTTCACTTGTACGAGACCTTC | Alternative oxidase 4 |
|  |  | R: CAGAGTTACCACCCAATGCTTCC |  |
| 6 | Cluster-21662.105304 | F: AAGGTGGCGAAGCAGGACAAG | Predicted: 40S ribosomal protein S30 |
|  |  | R: CGACGGCGGTGACGAAGC |  |
| 7 | Cluster-21662.82126 | F: TCGCACCCTGGACTCACAC | Ribosomal subunit 8E protein |
|  |  | R: CCCTCAAGAATGTAGCCATCAGC |  |
| 8 | Cluster-21662.104542 | F: AACGGCTTCTCCTCTTCCTACC | 60S ribosomal protein L32-1 |
|  |  | R: GCCCTCTTGAAGTGCTTGACC |  |
| 9 | Cluster-21662.86658 | F: CGTGTATGTTCTCCACCACCATTC | Beta-fructofuranosidase |
|  |  | R: GTAGGCAGTCCTGTACCTCTCG |  |
| 10 | Cluster-21662.85706 | F: CCGCTCGCTGTGCTCGTC | Flavonoid 3'-monooxygenase |
|  |  | R: CCGTGGCTGGACTGGATGG |  |
| 11 | Cluster-21662.92997 | F: CTCTCGGTCTCATCTCCTCCAG | Phenylalanine ammonia-lyase |
|  |  | R: TTGACAGCATTCTTGACATTCTCC |  |
