# Supplementary material for: Transcriptomic resources for prairie grass (Bromus catharticus): expressed transcripts, tissue-specific genes, and identification and validation of EST-SSR markers
Source: BMC Plant Biol. 2021 Jun 7;21:264. doi: 10.1186/s12870-021-03037-y (PMC8186225; doi:10.1186/s12870-021-03037-y)
Supplement: Supplementary file 10 — Additional file 10: Table S5. Morphological trait list of B. catharticus : names, abbreviations and measuring methods. [file 12870_2021_3037_MOESM10_ESM.docx]

**Table S5.** Morphological trait list of *B. catharticus*: names, abbreviations and measuring methods

| Traits | Abbreviations | Units | Measuring methods |
| --- | --- | --- | --- |
| Plant height | PH | cm | The absolute height from base to the top of plant (three reproductive tillers) |
| Stem diameter | SD | mm | The diameter of the second node of stem (three reproductive tillers) |
| Flag leaf length | FLL | cm | Length of the flag leaf, from ligule to tip (three reproductive tillers) |
| Flag leaf width | FLW | mm | Width of the flag leaf, at the widest position (three reproductive tillers) |
| Penultimate leaf length | PLL | cm | Length of the top second leaf, from ligule to tip (three reproductive tillers) |
| Penultimate leaf width | PLW | mm | Width of the top second leaf, at the widest position (three reproductive tillers) |
| Length of first internode | LFI | cm | Length between the first and second stem node from ground (three reproductive tillers) |
| Tiller number | TN |  | Tiller number for each plant |
| Dry matter yield | DMY | kg/plant | Air dry matter yield of the plant with stubble height at 6cm, when 80% seeds matured |
| Fresh matter yield | FMY | kg/plant | Fresh matter yield of the plant with stubble height at 6cm, when 80% seeds matured |
| Days from seeding to heading | DSH | day | Days from seeding to heading stage (heading plants account for 70% of all plants in the plot) |
